# Polyaxone monaxonids: revision of raspailiid sponges with polyactine megascleres ( *Cyamon* and *Trikentrion*)

**DOI:** 10.3897/zookeys.239.3734

**Published:** 2012-11-08

**Authors:** Rob van Soest, José Luis Carballo, John Hooper

**Affiliations:** 1 Naturalis Biodiversity Center, Dept. Marine Zoology, P.O.Box 9517 2300 RA Leiden, The Netherlands; 2 Instituto de Ciencias del Mar y Limnología, Universidad Nacional Autonoma de Mexico (Estación Mazatlán), Apartado postal 811, Mazatlán 82000, México; 3 Queensland Museum, P.O. Box 3300, South Brisbane, Queensland 4101, Australia & Eskitis Institute for Cell & Molecular Therapies, Griffith University, Australia

**Keywords:** Sponges, new species, revision, *Cyamon*, *Trikentrion*, polyactines, Raspailiidae

## Abstract

Among the thousands of non-tetractinellid (monaxonid) Demospongiae species, less than twenty possess polyactine (usually three- or four-claded) megascleres. These are currently assigned to two closely related genera, viz. *Cyamon* Gray and *Trikentrion* Ehlers, both members of the raspailiid subfamily Cyamoninae. The two genera are considered valid on account of differences in the shape and the ornamentation of the polyaxone spicules. *Cyamon* predominantly has four-claded equiangular spicules with all cladi spined or rugose, whereas *Trikentrion* usually has a majority of three-claded spicules on which spines are found only on a single basal clade. Nevertheless, the differences between the two genera appear to overlap in several known and newly discovered species, necessitating a revision of the two groups. Two new species of *Cyamon* were found to occur on inshore sandstone platforms off the coast of Mauritania. One of the new species, *Cyamon amphipolyactinum* **sp. n.**, possesses unique small ‘double’ polyactine spicules in addition to the usual calthrops-like polyactine megascleres characteristic for *Cyamon*. The second new species, *Cyamon arguinense* **sp. n.**,possesses polyactine megascleres of which only one of the cladi is spined the remaining three or more cladi being smooth, a feature that is considered characteristic of sponges of the genus *Trikentrion*. The type species of *Cyamon*, *Cyamon vickersii* (Bowerbank) appears to have been misinterpreted as a Caribbean species, because circumstantial evidence strongly indicates an Indian Ocean origin. This has the consequence that specimens recorded subsequently under the name *Cyamon vickersii* from various Western Atlantic localities are reassigned to *Cyamon agnani* (Boury-Esnault), a species originally described from Brazil. A new species, reported as *Cyamon vickersii* sensu Burton & Rao from the east coast of India, and available to us only as a single thick section mounted on a glass slide, is named *Cyamon hamatum* **sp. n.** The *Cyamon* membership of the only deep-sea species, *Cyamon spinispinosum* (Topsent) is drawn in doubt due to considerable morphological deviation from mainstream *Cyamon*. The type species of *Trikentrion*, *Trikentrion muricatum* (Pallas), is extensively described and discussed, and a neotype is assigned. West African *Trikentrion laeve* (Carter) is for the first time since its original description properly redescribed from the type material. The specimen recorded by Burton as *Trikentrion laeve* from Congo turned out to be different from the original material of Carter and is assigned to a new species, *Trikentrion africanum* **sp. n.** All species of both genera considered valid are reviewed, mostly based on the examination of type or other original specimens. Our revision shows the existence of twelve species of *Cyamon* and six species of *Trikentrion*. A key to the species is provided and remarks on the geographic distribution of both genera are made. Based on our study, the differences between *Cyamon* and *Trikentrion* are re-evaluated. Only one character absolutely distinguishes the two genera, the presence (*Trikentrion*) or absence (*Cyamon*) of trichodragmata. A further discriminating character is the possession of short thick styles (most *Cyamon* species) versus thick oxeas (many *Trikentrion*), but this is complicated by absence of the oxeas in three *Trikentrion* species. Although spination of the polyactine spicules in itself cannot serve to distinguish the two genera with certainty, those of *Trikentrion* are usually recognizable by excessive hook-like spines against a finer spination in *Cyamon*. Possibly, the polyactine spicules of both groups are non-homologous, with *Cyamon* polyactines derived from styles and *Trikentrion* polyactines from oxeas, but this remains to be further investigated.

## Introduction

The revision presented below was inspired by the recent discovery of two new species, evidently belonging to the sponge genus *Cyamon* Gray, 1867 (Demospongiae, Poecilosclerida, Microcionina, Raspailiidae, Cyamoninae), growing on shallow-water sandstone ridges off the coast of Mauritania. *Cyamon* species are unusual among raspailiid sponges in possessing polyactine megascleres (mostly four- or three-claded) with all cladi spined. Most species of *Cyamon* are rare encrusting sponges recorded from seemingly random localities across the warmer waters of the globe (Hooper 2002). Next to typical raspailiid ectosomal skeletal features, they share a plumose arrangement of smooth choanosomal styles and a basal mass of polyactines. To date ten species have been described (see Van Soest et al. 2012), usually recorded only once, from shallow waters of the Western Atlantic, Eastern Pacific, Indian Ocean and Indonesia, with a single species from deep-sea North Atlantic localities. A presumed sister genus, *Trikentrion* Ehlers, 1870, with only four species (see Van Soest et al. 2012), together occurring likewise circumglobally, has broadly similar polyactine spicules, with only one of the cladi spined. The two Mauritanian species were assigned to *Cyamon* because of the encrusting habit and stylote condition of the choanosomal megascleres. While one of them possesses unique and unprecedented ‘double’ micro-polyactines, it is the second species that appeared to be the most intriguing as it was found to possess polyactine spicules with only a single cladus spined, thus overlapping with the alleged spination in the polyactines of the sister genus *Trikentrion*. This raised the question whether the two genera could be part of a single diverse genus, rather than being separate morphological groups. *Cyamon* shares with members of the genus *Trikentrion* the polyactines and the raspailiid ectosome, but the choanosomal spicules in the type species *Trikentrion muricatum* and other *Trikentrion* species are smooth oxeas, and the polyactines in *Trikentrion* are scattered throughout the choanosome and replace the oxeas entirely in some species. The polyactines of *Cyamon* and *Trikentrion* appear distinctly different at first glance, with mostly equiangular geometry in *Cyamon* and sagittal (Y-shape, T-shape) condition in *Trikentrion*. Authors with experience of these sponges favour the hypothesis that the polyactine spicules derive from echinating acanthostyles, but there is no firm evidence for this and the spicule types remain unique in the family Raspailiidae and among the non-tetractinellid demosponges. Preliminary DNA sequence information confirmed the raspailiid affinity of at least *Trikentrion* (Erpenbeck et al. 2007), so there is at present no reason to take a different view.

Below, we describe four new species and review previously described species of both genera, pointing out aspects that appear to have been overlooked. We propose the synonymy of several previously accepted species, indicate a serious misinterpretation of the origin of the type species of *Cyamon* and provide extensive data on the type species of *Trikentrion*, including designation of a neotype. We demonstrate that the distinguishing characters of the two genera are eroded by intermediate conditions in new, but also in already known taxa, and discuss the remaining characters available for unambiguous genus assignment. We provide keys to the species and review the geographic distribution. We will refrain from taking decisions affecting the genus- and subfamily classification until such time that sufficient independent molecular support may become available. Recently, molecular evidence was presented that Raspailiidae, currently assigned to the suborder Microciona of the order Poecilosclerida (Hooper, 2002) is probably not closely related to the chela-bearing Poecilosclerida (Erpenbeck et al. 2007a; Morrow et al. 2012). While we acknowledge that this evidence will likely lead to alteration in the near future of the classification of the raspailiid sponges, including *Cyamon* and *Trikentrion*, we think it is currently premature to adopt these changes. More confirmation from additional studies and additional taxa is necessary to reassign Raspailiidae.

## Material and methods

Specimens of *Cyamon* and *Trikentrion* present in the collections of the Zoological Museum of Amsterdam and the Rijksmuseum van Natuurlijke Historie at Leiden (together now the Naturalis Biodiversity Center) were available from West Africa (two new species from a locality off Mauritania shown in Fig. 3, old collection specimens from Ghana), from the West Indian region (Curaçao and Colombia), the Seychelles, Indonesia and North Australia. We obtained loans of type material of most species from the collections of BMNH, USNM, MNHN, SMF, and LACM. One of us (JH) additionally examined fragments of *Cyamon vickersii* (Bowerbank, 1864) and *Trikentrion flabelliforme* Hentschel, 1912 obtained on loan from ZMB and SMF respectively. Non-type material of species of both genera was obtained on loan from BMNH and USNM (see below for abbreviations), and one of us (JLC) examined fresh material of *Cyamon koltuni* Sim & Bakus, 1986 and *Cyamon* (=*Trikentrion*) *catalina* Sim & Bakus, 1986. Details of collection numbers and localities are provided below with each species.

Abbreviations of institutions cited in the text:

**AHF-NHMLA** Allan Hancock Foundation, Natural History Museum Los Angeles County, USA

**BMAG** Bristol Museum and Art Galleries, Bristol, UK

**BMNH** British Museum of Natural History, London, UK

**LEB-ICML-UNAM** sponge collection Instituto de Ciencias del Mar y Limnología, Universidad Nacional Autonoma de Mexico (Estación Mazatlán), México

**MNHN** Muséum National d’Histoire Naturelle, Paris, France

**RMNH** Rijksmuseum van Natuurlijke Historie Leiden (now part of Naturalis Biodiversity Center)

**SMF** Senckenberg Museum, Frankfurt, Germany

**USNM** United States National Museum, Washington, USA

**ZMA** Zoological Museum Amsterdam (now part of Naturalis Biodiversity Center)

**ZMB** Zoologisches Museum Berlin, Germany

Terminology: We employ the collective word ‘polyactine’ for the spicules previously named acanthotriaenes by Hooper (2002) because the suffix –triaene suggests astrophorid affinities and also the triaene condition is only one of a range of cladi numbers in this spicule type (2–8). Other terms used in the literature (e.g. quadriradiates, cf. Carter 1879, acanthotetractine, cf. Hentschel 1912, pseudotetracts, cf. Dendy 1922; pseudactines, cf. Burton and Rao 1931; tetraxons, cf. De Laubenfels 1936) are equally unsuitable to capture the nature and variation of this spicule type. The cladi are subdivided into basal and lateral (see below). Furthermore, the style categories are indicated with the adjective ‘long thin’ for the extra-axial or peripheral long styles protruding from the surface and causing the hispidation of many species, ‘short thin’ for styles that form a bouquet or sheath around the long thin or thick styles in many species. In *Cyamon* we apply the term ‘short thick’ styles for the often subtylote styles that singly or in bundles form the choanosomal skeleton supported by the polyactines in many species. Spicules of *Trikentrion* are called ‘oxeas’ only when they represent choanosomal megascleres; reduced diactinal conditions of the polyactine spicules (also occurring in certain *Cyamon* species), recognizable by being roughened at one of the apices and usually swollen or crooked in the middle, are termed diactines or two-claded polyactines, not oxeas. Not all *Cyamon* and *Trikentrion* species appear to possess the full spicule complement of long thin, short thin and short thick styles/oxeas, so in individual species additional terms may be employed, notably ‘long subtylostyles’, which characterize *Cyamon quinqueradiatum* (Carter, 1880) and one of the new species. Several *Trikentrion* species lack choanosomal oxeas at all.

Microscopic preparation: dissoluted spicule preparations for measurements and SEM observations were made by dissolving a small fragment of the sponge in concentrated HNO_3_ or in undiluted household bleach, subsequent rinsing at least five times in distilled water, the last time in ethanol 96%, and finally pipetting a spicule suspension on stub or slide to be dried in a stove. Thick sections of the sponge made for the study of the skeletal structure were air-dried on a hotplate or in a stove and embedded in Canada balsam. Measurements of spicules (minimum-*average*-maximum) were made of 25 spicules of each category for each individual, unless otherwise stated (e.g. long thin spicules were often broken so the required number of spicules could not be measured).

## Results

We present the results in the following seven sections: a refined description and illustration of the type material of the type species of *Cyamon*, *Cyamon vickersii*, in which we argue that its original locality has been misinterpreted, followed by a description of recent (1993) Seychelles material considered to belong to *Cyamon vickersii*; description of two new *Cyamon* species from West Africa; descriptions and illustrations of all species assigned to *Cyamon* previously, including a new species based on misidentified material; a refined description of the specimens of the type species *Trikentrion muricatum* (Pallas, 1766) including assignment of a neotype; descriptions of the remaining species, including proposed synonymies and the description of a new species of *Trikentrion* based on misidentified material; we provide a key to the recognized species of *Cyamon* and *Trikentrion*; we make summary remarks on the geographic distribution of the two genera.

### Phylum Porifera. Class Demospongiae. Order Poecilosclerida. Suborder Microcionina. Family Raspailiidae. Subfamily Cyamoninae

#### Genus Cyamon

Gray, 1867

http://species-id.net/wiki/Cyamon

##### Type species:

*Dictyocylindrus vickersii* Bowerbank, 1864 (original designation).

##### Definition

**(emended):** Cyamoninae with skeleton consisting of a basal mass of polyactine spicules of which one or more cladi are spined or rugose in mature condition, supporting a plumose choanosomal skeletal arrangement of single or columnar groups of styles or subtylostyles with pointed ends outwards. Additional longer and shorter thin styles may be present in peripheral regions.

##### Remarks.

The styles are usually smooth, but in *Cyamon spinispinosum* (Topsent, 1904) both shorter and longer styles are spined (see below). In the type species, and several other species, thin short styles take the form of angulated and/or centrotylote strongylostyles, some of which have one end faintly or more heavily spined (see below). Polyactine spicules are genuinely polyaxone, with axial canals visible in all cladi. They are predominantly calthrops-like and have four cladi, but this may vary between two and eight cladi in some species. Usually, one of the cladi differs from the others by having a pointed spined apex, whereas the other cladi frequently have rounded ends, with prominent spined bulbs in several species, or they are occasionally entirely smooth, differing frequently also in length (either longer or shorter) from the other cladi. The spined pointed cladus is termed ‘basal’, under the assumption that it is homologous to the shaft of an ancestral echinating acanthostyle. The remaining cladi are here termed ‘lateral’, based on the assumption they are lateral proliferations of the acanthostyle head. One of the new species described below, has the polyactine spicules in two distinct categories, the smaller one of which is ‘amphipolyactine’ (see below).

*Trikentrion* Ehlers, 1870 shares the polyactines with *Cyamon*. According to the latest treatment of both genera (Hooper, 2002) the polyactines of *Cyamo*n would have all the cladi spined, whereas those of *Trikentrion* would have only the basal cladus spined. If this distinction between *Cyamon* and its close relative *Trikentrion* in the cladus spination would be maintained, then four species originally described as members of *Cyamon* would need to be transferred to *Trikentrion*, *Cyamon quinqueradiatum*, *Cyamon neon* de Laubenfels, 1930, *Cyamon argon* Dickinson, 1945 and *Cyamon catalina*, as well as one of the new species described below. We will demonstrate below and in the Discussion that cladus spination does not coincide with other more compelling differences with *Trikentrion* and consequently we will not transfer (all) the mentioned taxa.

The species considered valid members of *Cyamon* are listed in Table 1 and their properties in Table 2.

**Table 1.** Summary of taxonomic decisions on Cyamon and Trikentrion species

*Cyamon agnani* (Boury-Esnault, 1973 as Timea): valid species
*Cyamon amphipolyactinum* sp. n.: new species
*Cyamon argon* Dickinson, 1945: valid species
*Cyamon arguinense* sp. n.: new species
*Cyamon aruense* Hentschel, 1912: valid species
*Cyamon catalina* Sim & Bakus, 1986: transferred to *Trikentrion*
*Cyamon dendyi* De Laubenfels, 1936: j. syn. of *Cyamon vickersii*
*Cyamon hamatum* sp. n.: new species based on misidentified material of *Cyamon vickersii* sensu Burton and Rao 1931
*Cyamon incipiens* (Topsent, 1928 as Acantheurypon): j. syn. of *Cyamon spinispinosum*
*Cyamon koltuni* Sim & Bakus, 1986: valid species
*Cyamon neon* De Laubenfels, 1930: valid species
*Cyamon quadriradiatum* (Carter, 1880 as *Microciona*): species inquirenda
*Cyamon quinqueradiatum* (Carter, 1880 as *Microciona*): species inquirenda
*Cyamon spinispinosum* (Topsent, 1904 as *Hymeraphia*): valid species, atypical, possibly not a *Cyamon*
*Cyamon toxifera* Arndt, 1927: mixture of *Cyamon agnani* and *Clathria (Microciona) ferrea* (De Laubenfels, 1936)
*Cyamon vickersii* (Bowerbank, 1864 as *Dictyocylindrus*): valid species, type species, type locality proposed to be Indian Ocean, Central West Atlantic specimens transferred to *Cyamon agnani*
*Trikentrion africanum* sp. n.: new species, formerly *Trikentrion laeve* sensu Burton, 1948
*Trikentrion catalina* (Sim & Bakus, 1986 as *Cyamon*): valid species
*Trikentrion flabelliforme* Hentschel, 1912: valid species
*Trikentrion helium* Dickinson, 1945: valid species
*Trikentrion laeve* Carter, 1879: valid species
*Trikentrion muricatum* (Pallas, 1766 as *Spongia*): valid species, type species
*Trikentrion papillosa* (Sollas, 1879 as *Plectronella*): j. syn. of *Trikentrion muricatum*

**Table 2.** Summary of characters and spicule data of the species of *Cyamon* and *Trikentrion* considered valid in this study.

Genus	Species	Shape	height	Long thin style	Short thin style	short thin style centrotylote	Short thick style	Oxea	Polyactine cladi	Basal cladus	Lateral cladus	Tricho-dragmas
*Cyamon*	*Cyamon vickersii*	massive	30 mm	1700–2200 × 14–22	347–490 × 3.5–7	yes, spined	361–678 × 15–32	not present	3–5	54–102 × 9–18	39–78 × 7–16	not present
*Cyamon*	*Cyamon amphipolyactinum* sp.n.	encrusting	3 mm	1058–1643 × 6–12	288-456 × 2-4	no	204–558 × 9–33	not present	(1) 3–6 (2) 5–10	(1)21–51 × 3–10 (2) 18–30 × 1–4	(1)22–51 × 3–10 (2) 9–24 × 1–3	not present
*Cyamon*	*Cyamon arguinense* sp.n.	encrusting	2–3 mm	1229–1668 × 12–18	244–719 × 2.5–9	no	not present	not present	4–5	51–69 × 5–8	31–78 × 4–8	not present
*Cyamon*	*Cyamon agnani*	encrusting	3–5 mm	960–2065 × 7–9	210–658 × 1.5–4	no	174–489 × 7–21	not present	3–5	32–66 × 3–10	30–87 × 4–10	not present
*Cyamon*	*Cyamon aruense*	massive	30 mm	162–1760 × 9–16	302–426 × 1.5–4	yes	297–456 × 8–17	not present	3–5	48–84 × 5–11	29–54 × 4–8	not present
*Cyamon*	*Cyamon koltuni*	encrusting	1 mm	900–1400 × 5–7	not present	no	150–425 × 10–25	not present	3–6	35–66 × 5–10	35–66 × 5–10	not present
*Cyamon*	*Cyamon neon*	massive	20 mm	860–1290 × 6–10	191–306 × 1.5–3	yes, spined	270–468 × 14–24	not present	2–4	33–69 × 6–14	30–132 × 7–14	not present
Cyamon	Cyamonargon	arborescent	35 mm	960 × 15	210–348 × 3–4	yes, spined	350–593 × 15–42	not present	2–5	33–78 × 6–22	30-162 × 5–21	not present
*Cyamon*	*Cyamon quadriradiatum*	encrusting	not known	1042 × 41	347	yes?	not present ?	not present	4	76	76	not present
*Cyamon*	*Cyamon quinqueradiatum*	encrusting	3 mm	129–1989 × 3-33	492–698 × 3–5	no	129–1989 × 3–33	not present	4–5	45–93 × 4–11	31–51 × 3–7	not present
*Cyamon*	*Cyamon hamatum* sp. n.	encrusting	unknown	1300 × 30	272–355 × 2.5–5	yes, spined	421–604 × 16–31	not present	3–4	104–126 × 11–21	42–65 × 10–20	not present
*Cyamon*	*Cyamon spinispinosum*	encrusting	1 mm	not present	302–366 × 7–10	yes	657–822 × 32–38	not present	3–8	90–234 × 9–14	15–36 × 6–12	not present
*Trikentrion*	*Trikentrion muricatum*	arborescent	200 mm	not present	not present	no	not present	287–528 × 13–31	2–3	78–156 × 12–27	42–84 × 12 –27	57–102 × 4–18
*Trikentrion*	*Trikentrion laeve*	arborescent	45 mm	750–1062 × 4–9	234–433 × 0.5–2.5	no	not present	175–242 × 6–13	2–4	59–89 × 10–15	47–75 × 9–13	32–60 × 4–11
*Trikentrion*	*Trikentrion flabelliforme*	flabelliform or arborescent	60–260 mm	405–1034 x3–9	182–392 × 0.5–4	no	not present	135–340 × 5–22	2–4	96–123 × 10–17	51–84 × 9–17	35–88 × 6–12
*Trikentrion*	*Trikentrion helium*	bladed bush	70 mm	952–3393 x18–42	372–510 × 2.5–3.5	no	not present	not present	2–4	66–144 × 8–30	96–192 × 7–36	84–123 × 10–15
*Trikentrion*	*Trikentrion catalina*	flabelliform	150 mm	1400–5400 × 8–40	130–730 × 3–8	no	not present	not present	3–4	79–126 × 16–31	156–236 × 18–29	63–88 × 7–13
*Trikentrion*	*Trikentrion africanum* sp.n.	thin branch	65 mm	295–1394 × 9–24	192–358 × 2–3	no	not present	not present	2–3	27–96 × 11–21	33–121 × 9–19	49–61 × 5–11

### Description of the type material of the type species of *Cyamon*

#### Cyamon vickersii

(Bowerbank, 1864)

http://species-id.net/wiki/Cyamon_vickersii

Figs 1A–D , 2A–D

Unnamed spicule; Bowerbank 1862: 831, pl. 36 fig. 15 (West Indies?).
Dictyocylindrus vickersii Bowerbank 1864: 267, figure 234 (West Indies?); Carter 1879: 292, pl. 27 figs 5–8 (West Indies); Carter 1880b: 42.
Cyamon vickersii ; Gray 1867: 546 (West Indies); Dendy 1922: 108, pl. 4 fig. 4, pl. 16 fig. 5 (Seychelles).
Cyamon vickersi ; Thomas 1973: 26, pl. 1 fig. 14 (Seychelles); Van Soest 1994a: 71 (Seychelles); Hooper 2002: 498, Fig. 17.
Cyamon dendyi de Laubenfels 1936: 80.
Not: Trikentrion wickersi (sic); Topsent 1889: 4, figure 2A (Campeche Bank, Gulf of Mexico); Topsent 1894: 35 (corrected to *Trikentrion vickersi*) = *Cyamon agnani*.
Nec: Cyamon vickersi var. toxifera Arndt 1927: 149, pl. 2 fig. 9, text figure 10 (Curaçao) = mixture of *Cyamon agnani* and *Clathria (Microciona) ferrea*.
Nec: Cyamon vickersii ; Burton and Rao 1932: 355 (S India) = *Cyamon hamatum* sp. n.
Nec: Cyamon toxifera ; de Laubenfels 1936: 80 = *Cyamon agnani*.
Nec: Cyamon vickersi ; De Laubenfels 1936: 80 (Florida); Little 1963: 48 (Gulf of Mexico); Hooper 2002: 498, Fig. 17 = *Cyamon agnani*.
Nec: Cyamon vickersi ; De Laubenfels 1950 (Bermuda) = *Timea* sp.

##### Material examined.

HolotypeBMNH 1877.5.21.1887, dry condition, labeled *from Mr Vickers, Dublin, West Indies ?*

**Figure 1.**
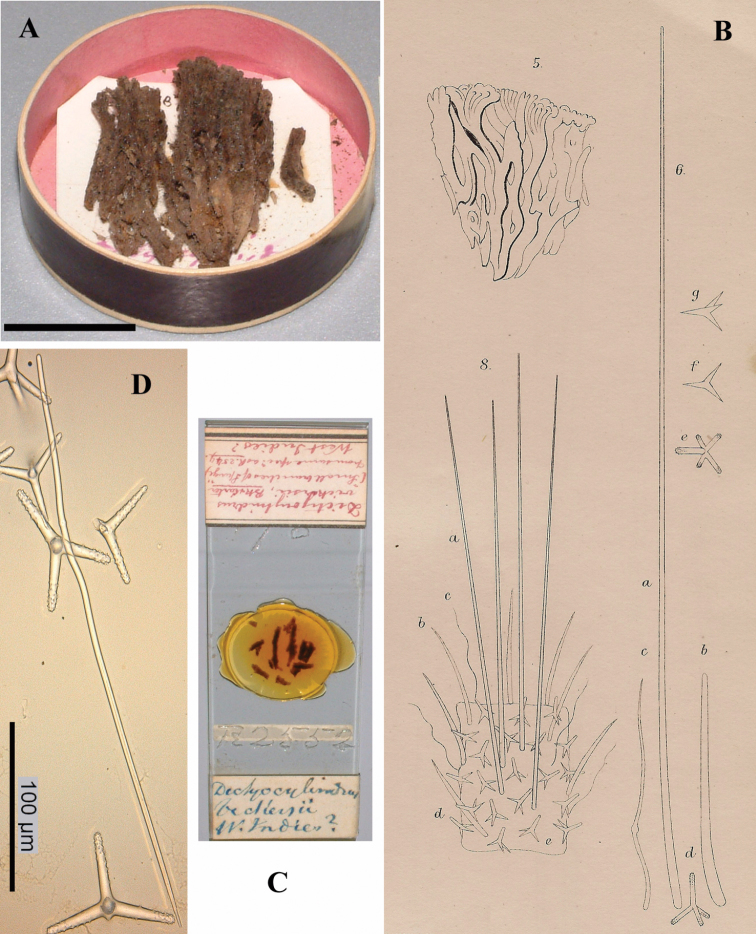
*Cyamon vickersii* (Bowerbank, 1864), holotype material, **A** holotype specimen BMNH 1877.5.21.188 (scale 1 cm) **B** illustrations from redescription of holotype by Carter (1879: plate 27 figs 5–6, 8) **C** photo of one of the original Bowerbank type slides containing thick sections **D** microphoto of spicules from one of the original Bowerbank type slides containing dissociated spicules.

The holotype was extensively described by Carter (1879) (his illustrations are reproduced in Fig. 1B), and redescribed by Hooper (2002). The specimen is now (2012, see Fig. 1A) a dry, macerated, wedge-shaped sponge, glued to a label containing the text *Bk. 1887, Dictyocylindrus vickersii*, lodged in a round box. There are five microscopic slides: three thick sections (one is reproduced in Fig. 1C), and two spicule mounts. A photo was made (Fig. 1D) of the contents of one of the spicule slides showing characteristic polyactines and one centrotylote strongylostyle. All microscopic slides are labeled with texts in Bowerbank’s and Carter’s handwritings.

##### Description.

The specimen consists of a barely coherent mass of columns, fragile, crumbly. Size approx. 3 × 2.5 × 0.6 cm. Colour now dark red-brown.

Skeleton: a branched columnar structure built by bundles of short thick styles supported at the base and along the column by masses of polyactines. The remaining spicules are not readily visible in the sections, so their positions are derived from Carter’s drawings (Fig. 1B): the columns are echinated by long and short styles and wavy strongylostyles.

**Figure 2.**
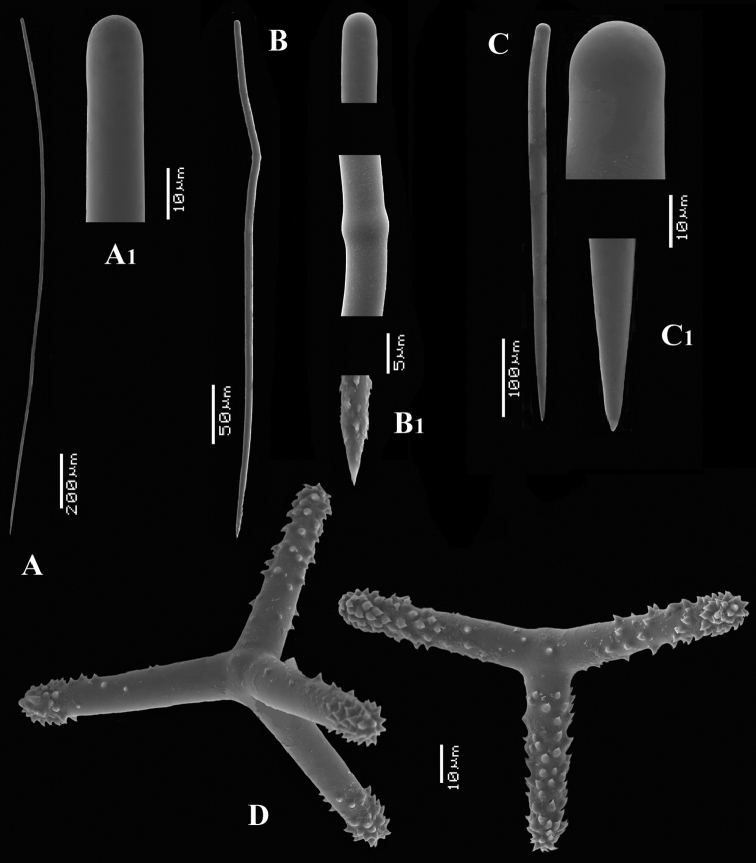
*Cyamon vickersii* (Bowerbank, 1864), SEM images of spicules of the holotype BMNH 1877.5.21.188, **A** long thin style **A1** details of apices of long thin style B short thin (strongylo-)style **B1**details of apices of short thin (strongylo-)style **C** short thick style **C1** details of apices of short thick style **D** four-claded (left) and three-claded (right) polyactines.

Spicules (Fig. 2): long thin styles, short thin (strongylo-)styles, short thick styles, polyactines.

Long thin styles (Fig. 2A, A1) curved, usually broken, rounded end faintly constricted subterminally, 1785–2200 × 14–22 µm.

Short, thin, crooked or wavy, centrotylote styles (Fig. 2B, B1), sometimes strongylote, with the pointed end often swollen or mucronate, and faintly to markedly spined, 355–*408.8*–490 × 3.5–*4.4*–6 µm.

Short thick styles (Fig. 2C, C1), smooth, curved subterminally at the rounded end, 470–*537.7*–662 × 15–*22.3*–32 µm.

Polyactines (2D), robust, mostly equiangular, predominantly four-claded, three-claded forms also rather common, five-claded spicules rare and much smaller than the other; juvenile spicules almost entirely smooth, mature spicules with all cladi spined at the ends, which are also lightly swollen; only sparingly spined near the centre; all cladi approximately equal in length, basal cladi barely distinct from lateral cladi: basal cladi 55–*62.5*–69 × 10–*12.6*–16 µm, lateral cladi 50–*65.6*–78 × 9–*12.4*–15 µm.

##### Remarks.

Contrary to most other authors referring to *Cyamon vickersii*, we have become convinced that this species does not occur in the Western Atlantic. The evidence for this is two-fold.

(1) There is considerable uncertainty about the origin of the type specimen. Bowerbank (1862: 831), when he first drew attention to the polyactine spicule, described it as follows:

*Spiculated inequi-angulated triradiate, with cylindrical entirely spined radii (Plate XXXVI. fig. 15). – From a fragment of a sponge presented to me by Mr. Vickers of Dublin*, who thinks it probably came from the West Indies.* This spiculum is an external defensive one. The triradiate rays are imbedded immediately beneath the dermal membrane, and the spicular ray is projected through it at right angles to its plane; they are very numerous*.

The part of the sentence we placed in roman lettering contains the only factual information on the origin of the specimen, which was subsequently named *Dictyocylindrus vickersii* by Bowerbank (1864: 267) with the same sentence and figure repeated. Bowerbank’s slides of the type material in BMNH marked as Bk 1887 were labeled prudently “West Indies ?” (see Fig. 1C), but first Gray (1867: 546) and later Carter (1879: 292) omitted the question mark. Carter did an extensive redescription of the Bowerbank material (see Fig. 1B), which properly established the characters of the species. Shortly before that (Carter 1876: 391) he alluded to a specimen with quadriradiate spicules obtained from Thomas Higgin from Grenada (Caribbean Sea), which he thought to belong to the same species. Higgin (1877: Pl. 14 Fig. 9) figured the spicule. However, both authors mentioned only long styles in addition to the polyactines, which is, as we know now, insufficient to characterize *Cyamon* species. As we described above, and was also clearly pictured by Carter himself (1879: Pl. 27 Fig. 6c, see also our Fig. 1B), *Cyamon vickersii* should possess undulated or crooked centrotylote thin styles or strongylostyles. We will demonstrate below that none of the Western Atlantic specimens of *Cyamon* we examined possess such spicules, in stead of which they have straight thin styles without centrotylote swelling or undulations. Nevertheless, from the time of Carter onwards it was assumed, that Bowerbank’s type came from the West Indies. Subsequent reports of *Cyamon* from Western Atlantic localities all employed the name *Cyamon vickersii*, and ignored the peculiar shape of the short thin styles.

**Figure 3.**
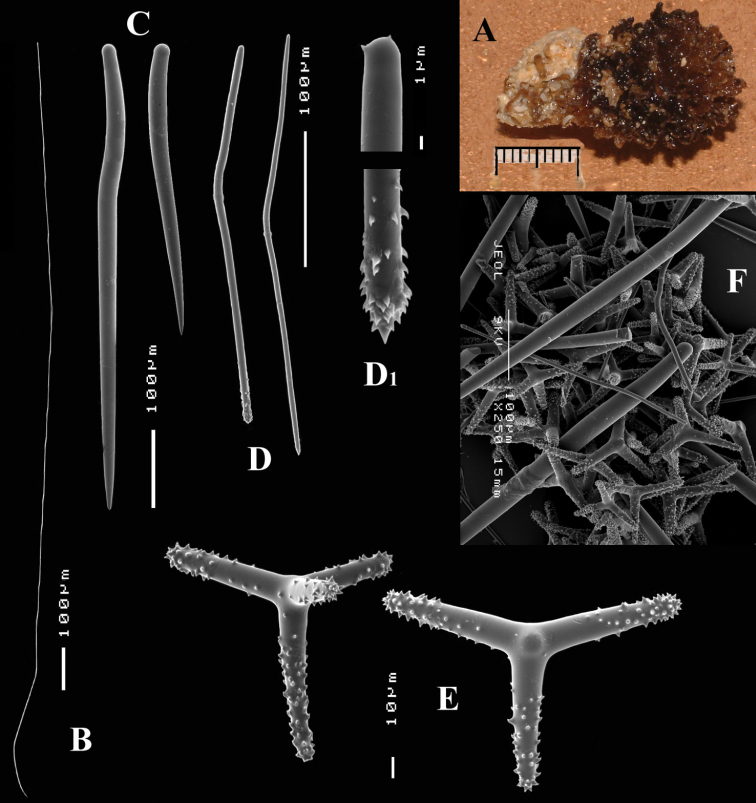
*Cyamon vickersii* (Bowerbank, 1864), ZMA material (Por. 10660) from the Seychelles **A** ‘strawberry’ shape (scale 1 cm) **B** long thin style **C** short thick styles **D** short thin (strongylo-)styles **D1** details of apices of short thin (strongylo-)style **E** polyactines **F** overview of spicules.

(2) Dendy (1922) and Thomas (1973) reported *Cyamon vickersii* from the Seychelles. Their descriptions exactly match the properties of Bowerbank’s type specimen, including the undulating short thin centrotylote styles. They especially mention the spination on the pointed ends of many of the undulating styles, precisely as we found in the type (see Fig. 2B, B1). De Laubenfels (1936: 80) also was of the opinion that the Seychelles material differed specifically from the Western Atlantic material. Because he believed that *Cyamon vickersii* was West Indian, he proposed the name *Cyamon dendyi* for the Seychelles material. Below, we describe and illustrate (Fig. 3) material obtained from the Seychelles, in which we demonstrate beyond doubt that it belongs to *Cyamon vickersii*.

To conclude: specimens identical or similar to the type of *Cyamon vickersii* are reported from the Seychelles. Specimens recorded from the Western Atlantic are dissimilar to the type of *Cyamon vickersii*, a.o. by lacking the characteristic undulating spicules. For the Atlantic representatives, the name *Cyamon agnani* (Boury-Esnault, 1973) is available (see below).

##### Description of ZMA material of *Cyamon vickersii*.

Figs 3A–F

##### Material examined.

Three samples, ZMA Por. 11729, preserved in alcohol, Seychelles, Amirante Islands, N of Poivre Island, 5.7333°S, 53.3333°E, Netherlands Indian Ocean Programme, Leg E, stat. 776/05, rectangular dredge, depth 43–48 m, coll. R.W.M. van Soest, 29–12–1992.

ZMA Por. 10660, preserved in alcohol, Seychelles, Amirante Islands, NE of D’Arros Island, 5.4S, 53.3167E, Netherlands Indian Ocean Programme, Leg E, stat. 750/09, rectangular dredge, depth 48–53 m, coll. R.W.M. van Soest, 26–12–1992.

ZMA Por. 12558, preserved in alcohol, Seychelles, N of Aride Island, 4.1833S, 55.6667E, Netherlands Indian Ocean Programme, Leg E, stat. 716/09, rectangular dredge, depth 40 m, coll. R.W.M. van Soest, 19–12–1992.

N.B.: Dendy’s (1922) specimen labeled and described as *Cyamon vickersii, BMNH 1931.1.1.19, Amirante, Sea Lark Expedition, 60 m*, was examined and photographed by J.H. (Hooper, 2002: Fig. 17) but could not be found in the collection of the Natural History Museum in 2011 (Ms Emma Sherlock, *in litteris*).

##### Description.

Strawberry-shaped sponge (Fig. 3A), forming a single semiglobular mass with microlobate surface. Color red or orange-red (alive), dark brown-red in alcohol. Consistency firm, barely compressible. Specimens now looking clathrate due to loss of thin surface membrane, still present in places. Size of largest specimen 3 × 2 × 2 cm.

Skeleton: condition described as columnar, consisting of hillock-like masses of polyactines, variable in thickness up to 2 mm, supporting thick plumose bundles of thick styles, which in turn are peripherally surrounded by short thin strongylostyles. Rare long thin styles are not present in all slides.

Spicules (Figs 3B–F): long thin styles, short thick styles, strongylostyles, polyactines, overview presented in Fig. 3F.

Long thin styles (Fig. 3B), very rare, invariably broken in small pieces, largest piece found in our slides 300 × 12 µm; according to Dendy they can reach 1700 × 14 µm. We reconstructed a long style from several pieces found on the SEM stub (Fig. 3B).

Strongylostyles (Figs 3D, D1), angulated, often faintly centrotylote, with unequal endings, smoothly rounded at one end, spined-mucronate at the other, 294–*347.1*–402 × 4–*5.6*–7 µm.

Short thick styles (Fig. 3C), characteristically curved in the upper half and provided with a faint tyle, shape of spicule fusiform, smooth, occasionally strongylote, 361–*538.9*–678 × 16–*24.1*–31 µm.

Polyactines (Fig. 3E), three- or four-claded in approximately equal proportions, a single five-claded form was observed in the slides (Dendy shows a reduced two-claded form). Basal cladi bluntly pointed, heavily spined apically, lightly spined along the shaft, lateral cladi ending rounded, equally heavily spined apically, less so along the shaft. In the center of the spicule there are usually no spines. Young growth stages are frequently entirely smooth. Basal cladi usually longer, 54–*77.5*–102 × 9–*14.4*–18 µm, than the lateral cladi, 39–*58.9*–78 × 7–*13.1*–16 µm, regardless of the number of cladi.

##### Distribution.

So far known with certainty from several localities throughout the Seychelles (Mahé and the Amirante Islands).

##### Ecology.

Sandy bottoms at 30–50 m surrounding reefs and atolls.

##### Discussion.

The ectosomal strongylostyles in *Cyamon vickersii* are reminiscent of those found in the type species of the Axinellidae genus *Reniochalina* (*Reniochalina stalagmitis* Lendenfeld, 1888), which Alvarez and Hooper (2009) suggested were indicative of a possible close relationship between *Reniochalina* and the Raspailiidae. This close relationship was further confirmed from molecular evidence (Erpenbeck et al. 2007b) showing affinities of *Reniochalina stalagmitis* with the raspaillid species *Axechina raspailioides* Hentschel (1912), indicating the strong morphological apomorphy of these ectosomal spicules for the Raspailiidae.

Burton and Rao (1932) reported *Cyamon vickersii* from South India (21 miles WSW from Mangalore), stating their specimen answered to Dendy’s (1922) material. We were able to examine a slide made by Burton (BMNH 1931.1.1.19a, the specimen is presumably in the collections of the Indian Museum), and found it to be close but nevertheless distinct from *Cyamon vickersii* proper. See below for a description and illustration, as *Cyamon hamatum* sp. n.

Gray’s (1867: 546) suggestion that the unnamed spicule without locality pictured in Bowerbank, 1864: figure 88 also belongs to *Cyamon vickersii* is debatable as the spicule with its single cladus spined conforms more likely to *Trikentrion*.

### Description of new species from Mauritania

#### Cyamon amphipolyactinum sp. n.

urn:lsid:zoobank.org:act:3AD5636E-F603-4011-9967-F2DF5D32350A

http://species-id.net/wiki/Cyamon_amphipolyactinum

Figs 4A–E , 5

##### Material examined.

**Type specimen**: HolotypeZMA Por. 22412, encrusting a stone, preserved in alcohol.

**Type locality**: Mauritania, off Banc d’Arguin, 19.0833°N, 16.4167°W, on sandstone ridge, dredged, 12–18 m. coll. R.W.M. van Soest & J.J. Vermeulen, Mauritania II Exped. Stat. 49, 11–06–1988.

##### Description.

**Figure 4.**
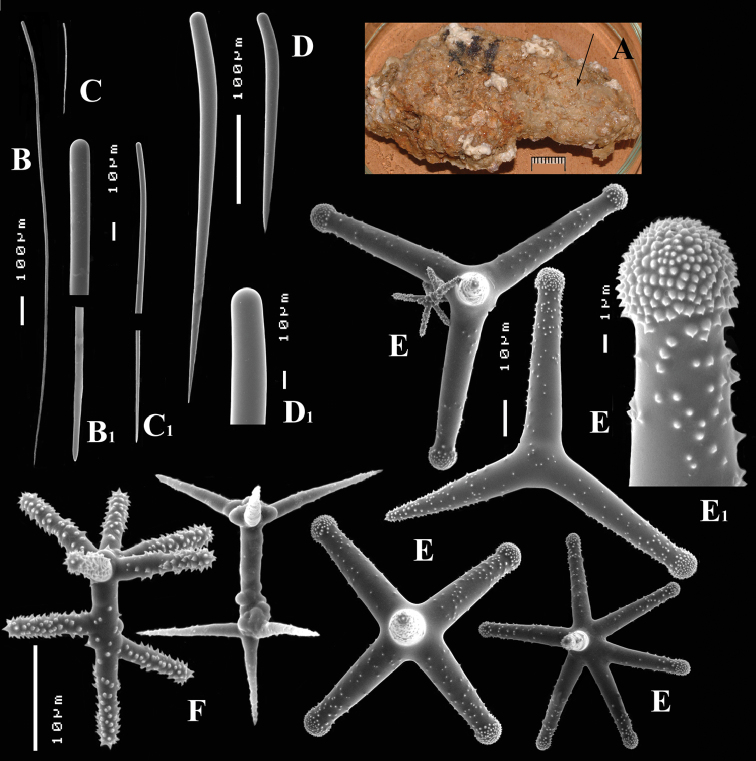
*Cyamon amphipolyactinum* sp. n., holotype ZMA Por. 22412, **A** shape (arrow) encrusting a fragment of sandstone (scale 1 cm) **B** long thin style **B1** details of apices of long thin style **C** short thin style **C1** details of apices of short thin style **D** short thick styles showing size variation **D1** detail of head of short thick style **E** polyactines (three-, four-, five-, and seven-claded) and one amphipolyactine showing size differences **E1** detail of bulbous end of lateral cladus **F** amphipolyactines full-grown and spined (left) next to incipient smooth spicule (right).

Encrusting a sandstone flake accompanied by several other encrustations (position of holotype indicated by arrow in Fig. 4A). Lateral size of holotype approximately 4x3 cm, thickness up to 3 mm. Color red in life, light orange brown in alcohol. Surface irregularly grooved and venous. Consistency soft, easily damaged.

Skeletal structure: A basal mass of polyactine spicules pierced by erect single or bundled thick styles, alternated by long thin styles protruding beyond the surface. At the periphery, the long styles are surrounded by bouquets of thin (tylo-)styles.

Spicules: of five types, long thin styles, short thin styles, short thick styles, large polyactines and small *double* polyactines.

Long thin styles (Figs 4B, B1), flexuous or curved snake-like, most were broken in the slides, size (based on 7 complete spicules): 1058–*1294.0*–1643 × 6–*9.3*–12 µm.

Short thin styles (Figs 4C, C1), curved, faintly tylote at the base, 288–*374.9*–456 × 2–*3.2*–4 µm.

Short thick styles (Figs 4D, D1), characteristically curved in the upper half, heads relatively thick with lower half narrowing strongly towards a sharp point, size varying strongly, 204–*352.1*–558 × 9–*17.4*–33 µm.

Large polyactines (Figs 4E, E1), in full-grown condition with all cladi ending in prominent, heavily spined knobs (Fig. 4E1) except one, the basal cladus, which is bluntly pointed. Cladi are less heavily spined towards the centre and at low magnification appear smooth. Growth stages may be partly or entirely without spines, but they are recognizable as unfinished by their irregularly undulating surface. The number of cladi varies between three and seven. In the largest spicules the cladi may be occasionally bifid. Basal cladi usually slightly shorter than the remaining cladi. Overall length of cladi regardless of condition is 18–51 × 3–10 µm.

Three-claded forms (rare), basal cladus 36–39 × 8–9 µm, lateral cladi 39–51 × 7–10 µm.

Four-claded forms (most common), basal cladus 18–51 × 3–9 µm, lateral cladi 22–51 × 3–9 µm.

Five-claded forms (also common), basal cladus 21–36 × 6–10 µm, lateral cladi 30–48 × 7–10 µm.

Six-claded forms (rare), basal cladus 21–36 × 4–5 µm, lateral cladi 24–38 × 4–6 µm.

Small *double* polyactines (Figs 4E and F), here termed amphipolyactines as they are obviously proliferated at both ends of the basal cladus. At first glance they resemble amphiasters or metasters (family Pachastrellidae Carter, 1875), but when studied with SEM they are similar in structure and ornamentation to the larger polyactines, but lack the swollen apices of the cladi of the larger ones. Cladi number from 5 to 10 (average 6.4) and they are spined in full-grown condition, smooth when still unfinished. Longest axis, presumably homologous to the basal cladus, is 18–30 × 1–4 µm, cladi 9–24 × 1–3 µm.

**Figure 5.**
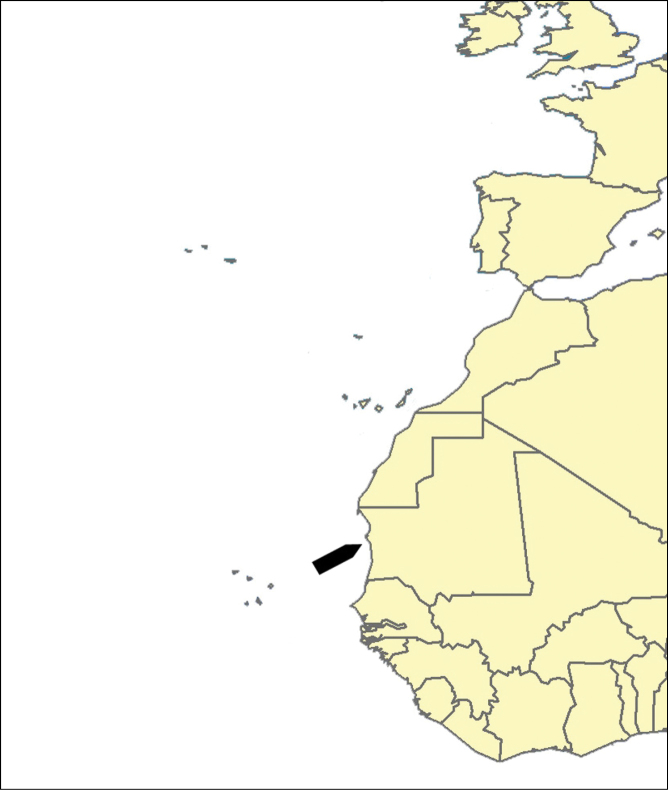
Map showing locality off the Mauritanian coast, where *Cyamon amphipolyactinum* sp. n. and *Cyamon arguinense* sp. n. were collected during the Netherlands Mauritania II Expedition, June 1988.

##### Etymology.

The name is an adjective that reflects the possession of unique small *double* polyactines, unprecedented in *Cyamon* and sponges in general.

##### Distribution

(Fig. 5). So far known only from the sandstone ridges of coastal flats of the Banc d’Arguin, Mauritania, West Africa.

##### Ecology.

In shallow-water (12–18 m), highly sedimented environments, in the company of many other sand dwelling sponges such as *Ciocalypta* Bowerbank, 1862 and *Polymastia* Bowerbank, 1864 (cf. Van Soest 1993: Pl. I fig. a).

##### Discussion.

The new species stands out among all described *Cyamon* and *Trikentrion* species by having unique *double* micro-polyactines. Further striking characters of the new species are the prominent heavily spined bulbous knobs of the large polyactines, which are only similarly developed in Californian *Cyamon koltuni* Sim & Bakus, 1986, and the high frequency of five-claded polyactines, which has been to that extent reported only for *Cyamon quinqueradiatum* (Carter, 1880) and *Cyamon koltuni*. The structure of the skeleton and the overall spiculation is shared with the type species of the genus, *Cyamon vickersii* and its close relative *Cyamon agnani*. Differences are the sizes of the spicules and the less prominent bulbous knobs on the cladi of the polyactines in the latter two species.

The remaining species appear more distinct with differences in the megascleres (apparent lack of thin styles in *Cyamon spinispinosum* and *Cyamon koltuni*), or the polyactine spicules (predominantly three cladi in *Cyamon neon* and *Cyamon argon*, smooth cladi except basal cladus in C. *quinqueradiatum* and *Cyamon arguinense* sp. n., irregular polyactines in *Cyamon spinispinosum*, lack of bulbous endings of the cladi and more densely overall spined in *Cyamon quadriradiatum* (Carter, 1880), and *Cyamon aruense* Hentschel, 1912).

#### Cyamon arguinense sp. n.

urn:lsid:zoobank.org:act:0024C5CC-3BBE-4043-93C7-22F2766B7E13

http://species-id.net/wiki/Cyamon_arguinense

Figs 5 , 6A–D

##### Material examined.

**Type specimen**: HolotypeZMA Por. 06723, encrusting a stone, preserved in alcohol.

**Type locality**: Mauritania, off Banc d’Arguin, 19.0833°N, 16.4167°W, on sandstone ridge, dredged, 12–18 m, coll. R.W.M. van Soest & J.J. Vermeulen, Mauritania II Exped. Stat. 49, 11–06–1988.

##### Description.

**Figure 6.**
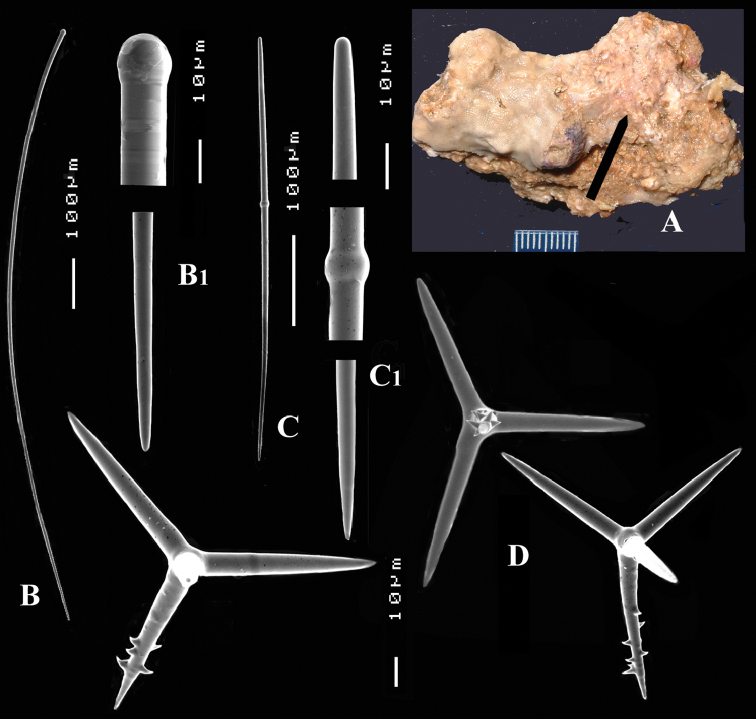
*Cyamon arguinense* sp. n., holotype ZMA Por. 06723, **A** shape (arrow) encrusting a fragment of sandstone (scale 1 cm) **B** subtylostyle **B1** details of apices of subtylostyle **C** short thin centrotylote style **C1** details of apices and middle part of short thin centrotylote style **D** polyactines.

Thin crust, (Fig. 6A) hispid surface. Color red (alive), dirty white (alcohol). Consistency soft, easily damaged, size 2.5 × 1.5 cm × 2–3 mm.

Skeleton: columnar bundles of megascleres issuing from a basal layer of polyactines. Columns consist of a single long subtylostyle sheathed in a tight bundle of fusiform centrotylote styles; bundles separate, interconnected only near the substratum.

Spicules of three types: subtylostyles (assumed to be homologues of the long thin styles), centrotylote styles (assumed homologues of the short thin styles), polyactines (short thick styles apparently lacking).

Long thin (subtylo-)styles (Fig. 6B, B1) with prominent heads, and bluntly rounded pointed ends, 1229–*1482.1*–1668 × 12–*13.9*–18 µm.

Short thin styles, fusiform, centrotylote (Fig. 6C, C1), tyle slightly excentric, rounded end tapering, 244–*521.5*–719 × 2.5–*6.4*–9 µm.

Polyactines, (Fig. 6D) predominantly four-claded, (a few five-claded forms were observed), basal cladus with coarse recurved spines, lateral cladi entirely smooth, basal cladus 51–*58.6*–69 × 5–*6.5*–8, lateral cladi 31–*55.7*–78 × 4–6.1–8 µm.

##### Etymology.

The name is an adjective referring to the type locality: the Mauritanian nature reserve Banc d’Arguin, one of the richest faunal areas of the west coasts of Africa (cf. Wolff et al. 1993).

##### Distribution

(Fig. 5). So far known only from the sandstone ridges of coastal flats of the Banc d’ Arguin, Mauritania, West Africa.

##### Ecology.

In shallow-water (12–18 m), highly sedimented environments, in the company of many other sand dwelling sponges such as *Ciocalypta* and *Polymastia* (cf. Van Soest 1993: Pl. I fig. a).

##### Discussion.

The single spined cladus of the polyactine spicules is an alleged feature of the genus *Trikentrion*, but in all other characters (growth form, monaxone spicules and skeletal arrangement) this is a typical *Cyamon*. It reminds strongly of Indian Ocean *Cyamon quinqueradiatum*, with which it shares the shape and upper length of the subtylostyles, the lack of differentiated long and short thick styles, and the size and single cladus spination of the polyactines. Differences are the predominantly five-claded polyactines and the shape and size of the stylote spicules in *Cyamon quinqueradiatum*. Long subtylostyles with prominent heads are shared with Indian Ocean *Cyamon quadriradiatum* but that species has all the cladi of the polyactines densely spined.

The new species was collected in the same dredge sample as *Cyamon amphipolyactinum* sp. n. (see above), but on a different sandstone flake (these provide hard substratum for sponges that would otherwise be buried in the sand). The two species differ sharply in the shape, size and ornamentation of the polyactines as well as in the shape and size of the styles.

### Descriptions of further species assigned to *Cyamon*

#### Cyamon agnani

(Boury-Esnault, 1973) comb. n.

http://species-id.net/wiki/Cyamon_agnani

Figs 7A–D , 8A–F

Hymeraphia sp.; Carter 1876: 391; Higgin 1877: 296, pl. 14 fig. 9 (Grenada)
Microciona quadriradiata Carter, 1880: 42 (in part, only what was illustrated in Higgin 1877).
Trikentrion wickersi (sic); Topsent 1889: 4, fig. 2A (Campeche Bank, Gulf of Mexico); Topsent 1894: 35 (corrected to *Trikentrion vickersi*).
Cyamon vickersi ; De Laubenfels 1936: 80 (Florida); Little 1963: 48 (Gulf of Mexico); Mothes et al. 2004: 6 (Brazil).
Cyamon vickersi var. toxifera Arndt 1927: 149, pl. 2 fig. 9, text figure 10 (Curaçao) = mixture of *Cyamon agnani* and *Clathria (Microciona) ferrea* (de Laubenfels, 1936 as *Fisherispongia*).
Cyamon toxifera ; de Laubenfels 1936: 80.
Timea agnani Boury-Esnault 1973: 276, fig. 24 (N.E. Brazil).
Not: Dictyocylindrus vickersii Bowerbank 1864; Carter 1879 = *Cyamon vickersii*
Nec: Microciona quadriradiata Carter 1880: 42 (in part: Gulf of Manaar specimen).
Nec: Cyamon vickersi ; De Laubenfels 1950 (Bermuda) = *Timea* sp.

##### Remark.

In view of the proposed major change in the status of *Cyamon* specimens reported from the Western Atlantic, description of the available material is presented in two sections, first the holotype of *Cyamon agnani*, subsequently other specimens known from the area and proposed to be assigned to *Cyamon agnani*.

##### Description of MNHN holotype.

Figs 7A–D

##### Material examined.

HolotypeMNHN NBE 947, preserved in alcohol, Brazil, NE coast, Calypso stat. 97, 21.1667°S, 40.7°W, 12 m depth.

##### Description.

Small hispid crust, color ochre. Detachable skin. The material borrowed from MNHN measured a few mm^2^ encrusting a small piece of coral.

Skeleton: basal layer of polyactines, upon which megascleres are erected individually.

Spicules: long thin styles, short thick styles, polyactines.

**Figure 7.**
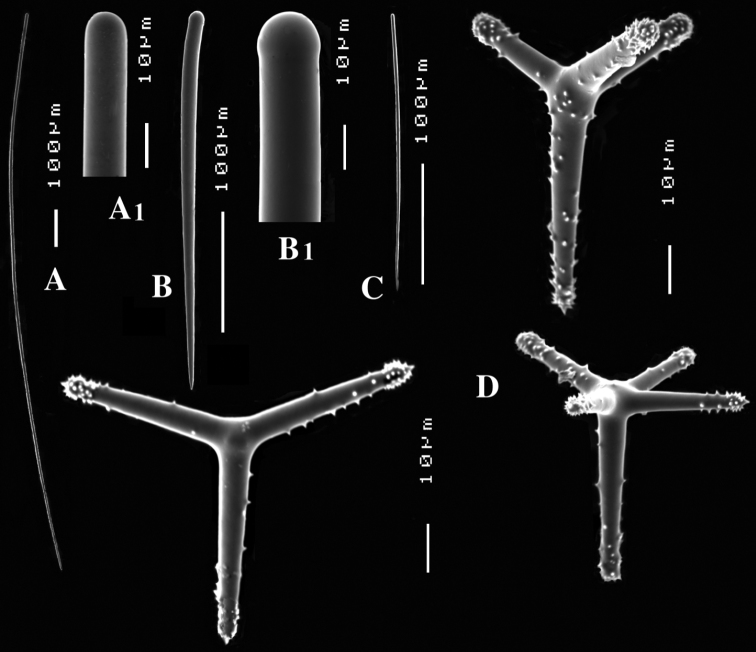
*Cyamon agnani* (Boury-Esnault, 1973), holotype MNHN NBE 947, **A** long thin style **A1** detail of head of long thin style **B** short thick style **B1** detail of head of short thick style **C** short thin style **D** polyactines.

Long thin styles, curved, variable in length, possibly in two size categories, but difficult to establish due to broken condition of most spicules, longest complete spicule 960 × 7 µm (Fig 7A).

Short thin styles were not mentioned in Boury-Esnault (1973), but there were a few small broken styles and one complete spicule measuring 210 × 4 µm (Fig. 7C).

Short thick styles (Fig. 7B), curved in the upper half, ending in a slight tyle, smooth, slightly variable in length and thickness, 183–*236.7*–315 × 7–*9.3*–12 µm.

Polyactines (Fig. 7D), with three to five cladi (usually four), cladi lightly spined along the shaft but with heavily spined endings, with a blunt ending in the basal cladus, and slightly inflated rounded endings in the lateral cladi. Basal cladi 32–*38.5*–48 × 3–*4.8*–7 µm, similar sized lateral cladi, 30–40 × 5 µm.

**Figure 8.**
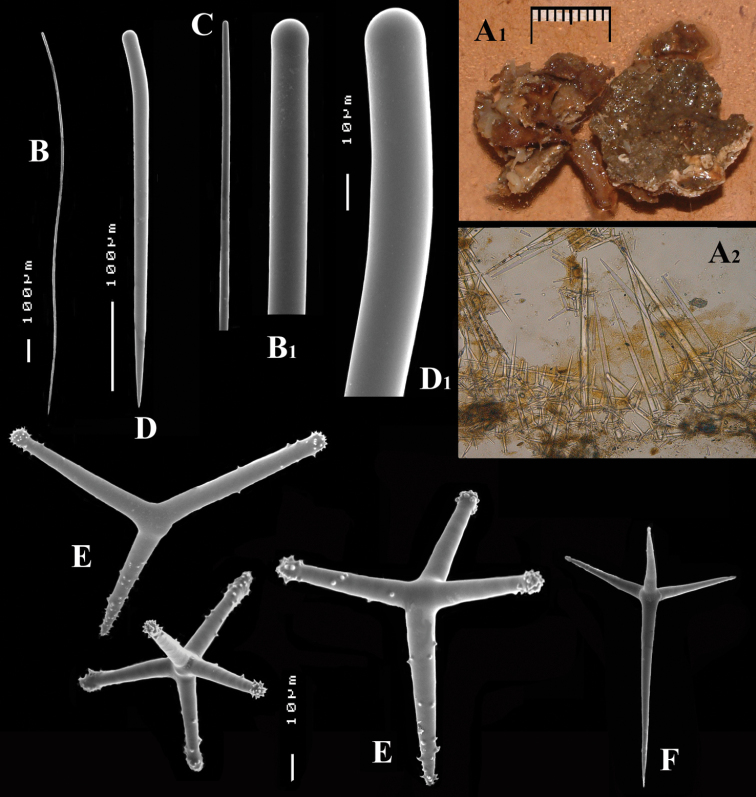
*Cyamon agnani* (Boury-Esnault, 1973), ZMA Por 10539 from NE Colombia **A1** shape (scale 1 cm) **A2** cross section of skeleton **B** long thin style **B1** detail of head of long thin style **C** upper part of short thin style **D** short thick style **D1** detail of head of short thick style **E** polyactines **F** incipient polyactine showing smooth cladi.

##### Discussion.

The *Cyamon* nature of this material was previously detected by Mothes et al. (2004), who examined the present type material. Their conclusion was corroborated by Van Soest (2009) in his discussion of *Timea* species of the West Atlantic region. Mothes et al. (2004) proposed to assign *Timea agnani* to the synonymy of *Cyamon vickersii*, but as explained above, that species differs in spiculation and geographic distribution. Despite the scanty available type material and the poor representation of short thin styles, it looks as if the categories, sizes and shapes of the spicules are broadly similar between the type of *Cyamon agnani* and Caribbean and Carolinian specimens recorded as *Cyamon vickersii* (see for details below). It is proposed here to consider all these Western Atlantic specimens as members of a widespread *Cyamon agnani*.

##### Description of ZMA material and discussion of further Western Atlantic records. 

Figs 8A–F

##### Material examined.

ZMA Por. 00828, holotype of *Cyamon vickersii* var. *toxifera*, preserved in alcohol, from Curaçao, Spaanse Water, on dead *Porites* coral, 12.076N, 68.858W, coll. C.J. van der Horst, field number 65a, 19–05–1920.

ZMA Por. 10539, preserved in alcohol, Colombia, Santa Marta region, El Morro, 15 m, 11.25N, 74.2167W, coll. B. de Jongh, 26–10–1989 (Fig. 1A2).

USNM 22456, preserved in alcohol, Florida, SE of Loggerhead Key, on a block of limestone dredged from 70 m, coll. M.W. de Laubenfels, 26 June 1932.

USNM 221078 (23563), preserved in alcohol, Florida, Northern Gulf of Mexico, Apalachee Bay, rock and sand, 29.785 – 29.8°N, 84.325°W, 11 m, coll. F. Little, 1956-57;

USNM 33518, preserved in alcohol, off South Carolina, RV *Oregon* (S.C. Mar. Res. BLM), stat. 0SO6, 32.4883°N, 78.8217°W, 48 m, collected by grab, 4 May 1981.

##### Description.

(Based on ZMA Por. 10539). Irregular encrustation (Fig. 8A1), with hispid, bumpy surface (preserved condition). Size 3 × 2.5 cm in lateral expansion, 3-5 mm in thickness. Colour (alive) red, (alcohol) red-brown. Consistency soft.

Skeleton (Fig. 8A2): basal mass of polyactine spicules penetrated by single short thick styles erect with heads embedded in the substrate. Long thin styles also erect on the substrate with rare short thin styles arranged around the peripheral protruding apices. This ‘raspailid’ feature was only observed in a few places.

Spicules: long thin styles, short thin styles, short thick styles, polyactines.

Long thin styles (Figs 8B, B1), complete ones with a wavy outline (Fig. 8B), but mostly broken in the slides, largest complete style 2065 × 9 µm, with smaller pieces varying down to 1170 × 7 µm.

Short thin styles, straight (Fig. 8C), 423–*486.6*–658 × 2–*2.2*–2.5 µm. We were unable to find a complete spicule on the SEM stub, so we only show a broken spicule in Fig. 8C.

Short thick styles, (Figs. 8D, D1) curved in the upper half, with a faint tyle, smooth, in a large size range, 174–*358.2*–489 × 9–*14.4*–21 µm.

Polyactines (Figs 8E–F), with three to five cladi (usually four), typically with all cladi mostly smooth but ending in a spined apex, the basal cladus usually bluntly pointed, the lateral cladi with inflated endings (Fig. 8E), early growth stages smooth and with all cladi pointed (Fig. 8F), cladi often of unequal length but without clear pattern of variation, basal cladi 39–*56.4*–66 × 6.5–*8.3*–10 µm, either longer or shorter than the lateral cladi, 36–*61.6*–87 × 4.5–*7.6*–10 µm.

##### Distribution.

Greater Caribbean, Gulf of Mexico, South Carolina, N.E. Brazil.

##### Ecology.

Encrusting dead corals and other limestone substrates, 0–70 m.

##### Discussion.

Topsent (1889) records thinly encrusting specimens of the species under the name *Trikentrion wickersi*. This was apparently a common species on the Campeche Bank in the Mexican part of the Gulf of Mexico. His specimens were violet or blackish brown in color (preserved) and he observed that next to four-claded spicules also five-claded and three-claded occurred, though rarely. His drawings of the polyactines conform closely to those of our material, but no spicule sizes were given. Topsent (l.c.) believed that the similarities between *Cyamon* and *Trikentrion* were too great to keep them as separate genera, but his choice of *Trikentrion* as the valid name for the group is incorrect as *Cyamon* is the older name.

De Laubenfels (1936: 80) recorded the species from Florida from a depth of 70 m as a bright orange crust with lateral expansion of 7 cm^2^ and thickness of 1 mm. This specimen, USNM 22456, which was received on loan from the Smithsonian Insitution, showed long thin styles up to 2 mm (one complete spicule measured 1939 × 9 µm); short, straight, thin styles 270–590 × 1.5–3 µm (not mentioned by De Laubenfels); short thick styles 420–602 × 27–32 µm (also not mentioned by De Laubenfels); polyactine spicules (three-, four- and five-claded) with basal cladi 51–63 × 9–14 µm and lateral cladi 39–51 µm.

De Laubenfels (1950: 68, fig. 30) also reported the species from Bermuda (as *Cyamon vickersi*), depth not given. The specimen was probably not a *Cyamon*, because the drawings of the polyactine spicules appear to be rather those of a *Timea* aster with proliferated rays. The Bermuda occurrence must thus be considered suspect.

Little (1963) recorded *Cyamon vickersii* as an orange encrustation from the Gulf of Mexico, depth 11 m. His description is obviously copied from De Laubenfels (1936), as he gives exactly the same measurements of the spicules and also omitted to mention the short thick styles. We were able to examine this specimen, USNM 221078, thanks to a loan from the Smithsonian Institution. It has long thin styles 1050–1563 × 9 µm, short thin styles 330–345 × 2–3 µm, short thick styles 270–332 × 13–20 µm, polyactines (three-, four-, and five-claded) with basal cladi 36–60 × 7–12 µm and lateral cladi 33–61 × 7–10 µm.

The loan from the Smithsonian also included an undescribed specimen from South Carolina, USNM 33518. This had long thin styles of up to 2 mm, short thin styles 360–426 × 2–2.5 µm, short thick styles 410–500 × 22–23 µm, and polyactines (three- and four-claded) with basal cladi 48–93 × 12–15 µm and lateral cladi 45–49 × 12–14 µm.

Alcolado (1994) in an unpublished list of Cuban sponges lists *Cyamon vickersii* from Cuban waters, which presumably concerns also the species we here propose to call *Cyamon agnani*.

We investigated the type material of *Cyamon vickersii* var. *toxifera* Arndt, 1927 (the name should be corrected to *toxiferum* to match the gender of the genus), ZMA Por. 00828, from Spaanse Water, Curaçao, and discovered that the toxas forming the basis of Arndt’s variety are clearly foreign. They form part of the spiculation of a microcionid sponge, readily identified as *Clathria (Microciona) ferrea* (De Laubenfels, 1936 as *Fisherispongia*) by its characteristic polytylote subtylostyles (see also description of Curaçao material of that species in Van Soest 1984). This discovery means that the name *Cyamon (Microciona) ferrea* is threatened by Arndt’s variety. The material is so scanty, that any trace of *Cyamon* polyactines has now (2012) disappeared from the sample. De Laubenfels (1936: 80) elevated Arndt’s variety to specific rank; needless to say that this is unwarranted.

The spicule complement and the shape of the polyactines is broadly similar in the Brazilian type of *Cyamon agnani* and specimens recorded from Caribbean and Carolinean waters as *Cyamon vickersii*, but the latter may have long thin styles up to twice as long. The short thick styles and the polyactines also are on average clearly longer and more robust in Caribbean specimens. The geographic separation caused by the Amazonian outflow could be a barrier to gene flow between these shallow-water sponges, and the differences may thus have a genetic background. On the other hand, the Brazilian type material is only a single small specimen and variation in Brazilian waters may turn out to be as large as that in the Caribbean. Thus distribution and ecology for this species may be summarized as: tropical waters of Brazil, the Greater Caribbean and Gulf of Mexico, South Carolina, known from 0–70 m depth, usually encrusting dead corals and other limestone substrates.

#### Cyamon aruense

Hentschel, 1912

http://species-id.net/wiki/Cyamon_aruense

Figs 9A–E

Cyamon aruense Hentschel 1912; 374, pl. 20 fig. 33 (Aru Islands, Indonesia); Hooper 1991: 1305, figs 63f-i.

##### Material examined.

Fragment of holotypeSMF 1618, preserved in alcohol, Indonesia, Aru Islands, Straits of Dobo, 6°S, 134.8333°E, 40 m, coll. H. Merton, 20–03–1908.

##### Description.

**Figure 9.**
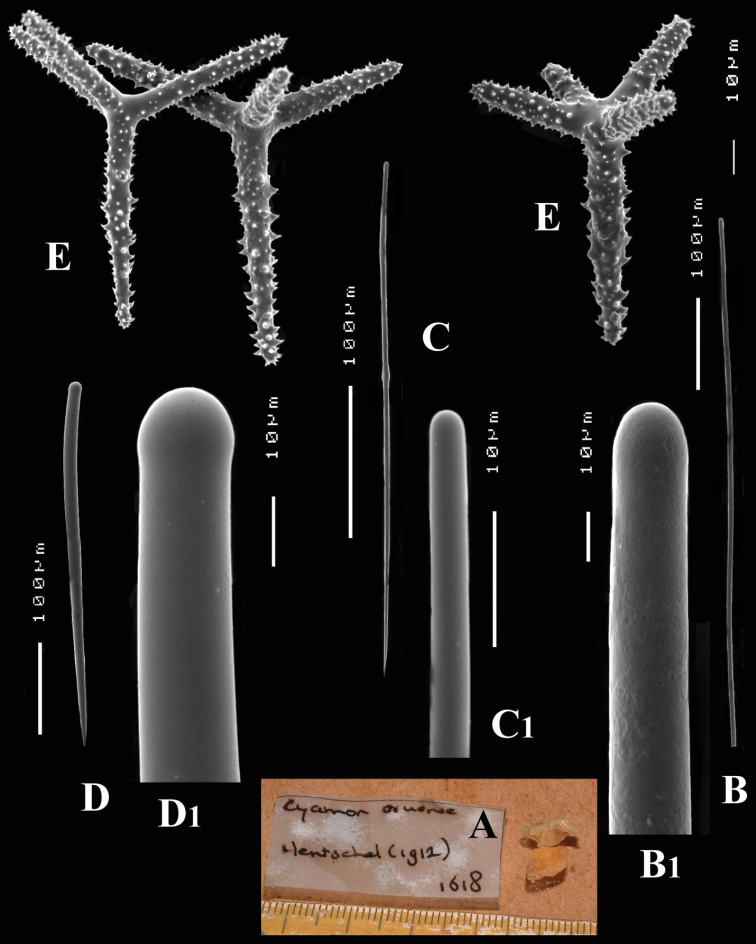
*Cyamon aruense* Hentschel, 1912, holotype SMF 1618, **A** fragments from holotype **B** long thin style (broken) **B1** detail of head of long thin style **C** short thin style **C1** detail of head of short thin style **D** short thick style **D1** detail of head of short thick style **E** polyactines.

The holotype is an encrusting sponge of 6 cm long and 3 cm wide growing over a haplosclerid sponge (Hentschel, 1912). The fragment of less than 0.5 × 0.5 cm and 1 mm in thickness (see Fig. 9A) examined by us was mixed with the haplosclerid in such a way that the microscopic slides were thoroughly contaminated with it. We have to rely on Hentschel’s remarks about shape and surface characters. The surface is hispid due to the long styles protruding from the sponge, which was grey coloured in alcohol, but shows a pale brownish colour in our fragment. Consistency not mentioned by Hentschel, but crumbly describes it best.

Skeleton: the usual basal mass of polyactinal spicules upon which relatively long styles are erected surrounded in the periphery by bundles of thin centrotylote styles. Thick short styles are singly erect on the substrate, buried in the basal mass of polyactines.

Spicules: long thin styles, centrotylote thin styles, short thick styles, polyactines.

Long thin styles (Figs 9B, B1), relatively rare, smooth, almost always broken in the slides so we cannot show a complete SEM image of them, heads smooth and not distinguished in width from the shaft, the other end gradually pointed. Longest style approximately 1620 × 16 µm, whereas Hentschel mentioned 1760 × 9–12 µm. Hentschel suggested a faint tyle, but we did not observe this.

Centrotylote thin styles (Fig. 9C, C1), smooth, curved, with a tyle near the middle of the spicule, but not exactly in the middle, the most common spicule of the monaxone spicule complement, 302–*368.7*–426 × 1.5–*2.6*–4 µm.

Short thick styles (Fig 9D, D1), relatively rare, smooth, often curved in the upper half, slightly fusiform, with a faint tyle, 297–*389.8*–456 × 8–*13.9*–17 µm.

Polyactines (Figs 9E) with 3-5 cladi, all of which are heavily spined with relatively coarse spines, without smooth areas, basal cladi rather blunt compared to those of other species, 48–*68.9*–84 × 5–*8.1*–11 µm, lateral cladi 29–*40.6*–54 × 4–*6.7*–8 µm.

##### Distribution.

Only known from the Arafura Sea.

##### Ecology.

Deeper water on hard substrate.

##### Discussion. 

The heavy spination of the polyactines appears to be a distinct feature of this species. Hooper’s (1991) redescription denies the occurrence in this species of centrotylote ectosomal thin styles, wheras these spicules appeared common in the fragment of the holotype examined by us. These spicules are comparable to those of *Cyamon arguinense* sp. n., rather than to those of *Cyamon vickersii* because they do not have the characteristic crooked shape and also are not rugose at the pointed end. The polyactines of this species appear somewhat similar to those of *Cyamon quadriradiatum* as described and drawn by Carter (1880). However, details and sizes of the other spicules differ between the two: long styles are much longer and thinner in *Cyamon aruense* and there is apparently no further category of short thick styles in *Cyamon quadriradiatum*. Since both are ill known, we must have more data and further specimens to establish these species as distinct.

#### Cyamon koltuni

Sim & Bakus, 1986

http://species-id.net/wiki/Cyamon_koltuni

Figs 10A–F

Cyamon koltuni Sim and Bakus 1986: 18, fig. 3 (California); Lee et al. 2007: 210.

##### Material examined.

HolotypeUSNM 33630, preserved in alcohol, California, Santa Catalina Island, Big Fisherman’s Cove, 33.45°N, 118.4833°W, 6 m.

LEB-ICML-UNAM 1497, preserved in alcohol, Mexican Pacific, Islas Marietas (Nayarit), Cueva Marietas, 20.7003°N, 105.5658°W, 11 m, coll. J.L. Carballo, 11–10–2006.

**Figure 10.**
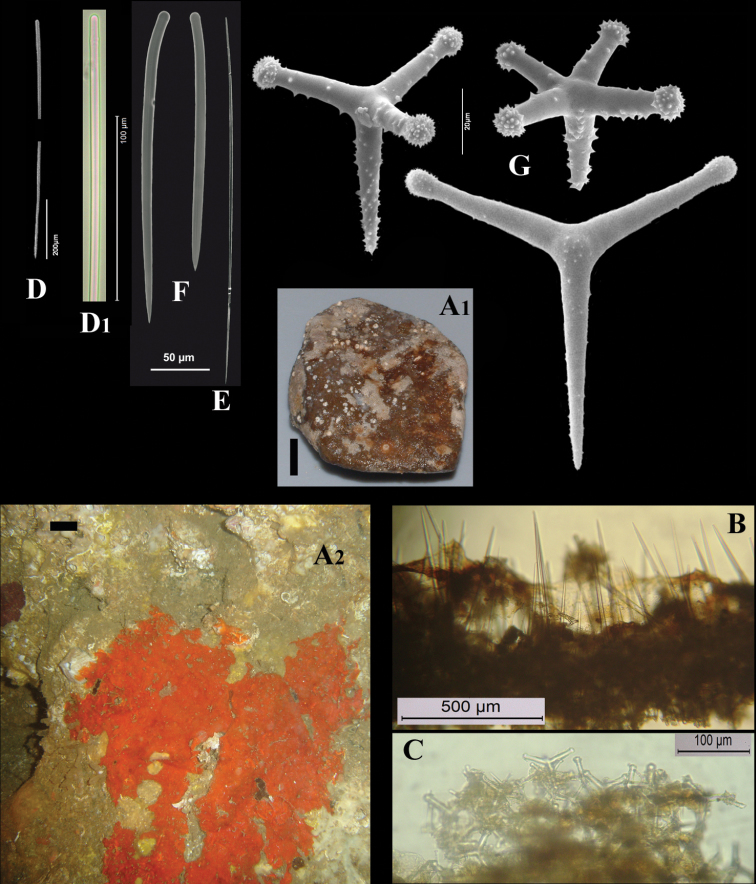
*Cyamon koltuni* Sim & Bakus, 1986, **A1** Californian holotype, USNM 33630, encrusting a rock (scale = 1 cm) **A2** Mexican Pacific specimen LEB-ICML-UNAM 1497 **B** cross section of peripheral region **C** thick section of basal mass of polyactines **D** fragments of long thin style **D1** microphoto of detail of rounded apex of long thin style **E** short thin style **F** short thick styles **G** three- to five-claded polyactines showing prominent bulbous ending of lateral cladi.

The holotype (Fig. 10A1) was received on loan from the Smithsonian Institution, but in view of the small crust and previous studies of it, including SEM examination (Sim and Bakus 1986: Fig. 3; Lee et al. 2007), and the presence of additional material, it was decided that no further sampling of it was necessary. We report the occurrence of a second specimen of this species from Mexican Pacific waters, from which we obtained our data for the description below.

##### Description.

Thinly encrusting (Fig. 10A1, A2) on rocks, color bright orange. Size of Mexican specimen 12 × 15 cm, thickness 1 mm. Surface very hispid.

Skeleton: a basal mass of polyactine spicules (Fig. 10B), upon which with styles are erected (Fig. 10C), no discernible skeletal organization due to thinness.

Spicules: long thin styles, short thin styles, short thick styles, polyactines.

Long thin styles (Fig. 10D, 10D1): rather straight, with faint subterminal tyle at the rounded end, 900–*967*–1400 × 5–*5.9*–7 µm.

Short thin styles (Fig. 10E), occasionally oxea-like with tapering thin endings, 265–370 × 2.5–5 µm.

Short thick styles (Fig. 10F): slightly curved and thickest subterminally near the faintly constricted rounded end, 150–*316*–425 × 10–*14.7*–25 µm.

Polyactines (Fig. 10G): three-six claded, cladi spined predominantly at the apices; basal cladi pointed, spined more heavily than the lateral cladi, which are provided with prominent bulbous apices, 35–*46*–66 × 5–*8.9*–10 µm.

##### Distribution.

Southern California, Pacific coast of Mexico.

##### Ecology.

Under rocks and in caves in shallow water.

##### Discussion.

The enhanced bulbous endings of the polyactines is distinctive and is only matched by those of *Cyamon amphipolyactinum* sp. n., but that species differs clearly by possessing a smaller category of amphipolyactines. It is generally similar to *Cyamon agnani*, differing from that species in the sizes of the styles and the very prominent bulbous endings of the cladi of the polyactines.

#### Cyamon neon

De Laubenfels, 1930

http://species-id.net/wiki/Cyamon_neon

Figs 11A–H

Cyamon neon De Laubenfels 1930: 28 (California); 1932: 109, fig. 65; Sim and Bakus 1986: 17 (California, with erroneous size data of the polyactines); Luke 1998: 10 (La Jolla, S California); Lee et al. 2007: 211.

##### Material examined.

Holotype USNM 21412, preserved in alcohol, California, between Point Dunes and Newport, near San Pedro.

**Paratype:** BMNH 1929.9.30.5, two slides, Santa Catalina Island, California, 33.5°N.

##### Description.

**Figure 11.**
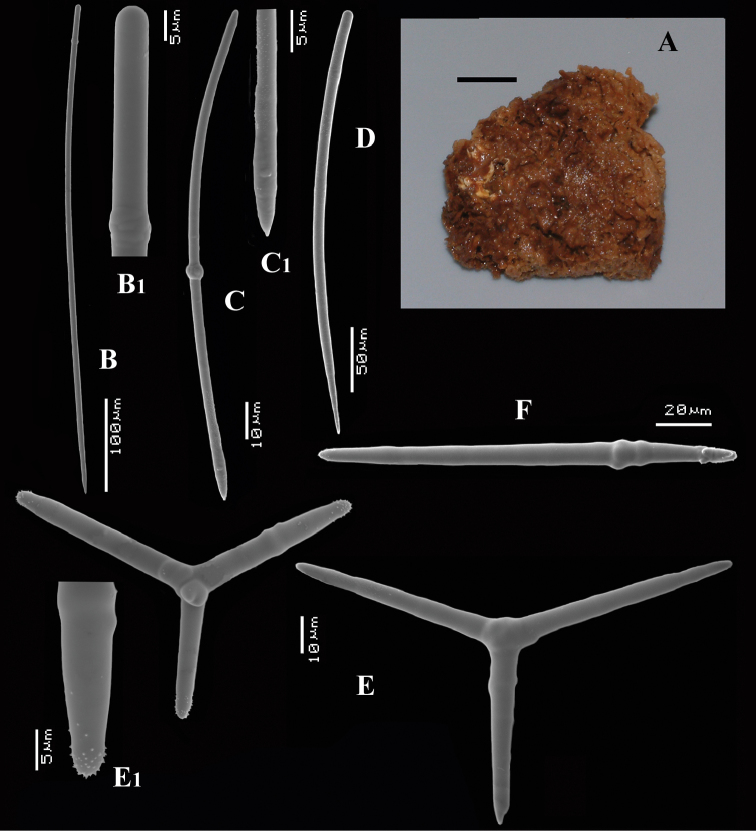
*Cyamon neon* De Laubenfels, 1930, holotype USNM 21412, **A** massively encrusting shape with irregular surface (scale = 1 cm) **B** long thin style **B1** detail of rounded end showing subterminal tyle **C** short thin centrotylote (strongylo-)style **C1** detail of swollen roughened apex of short thin (strongylo-)style **D** short thick style **E** polyactines **E1** detail of basal cladus of polyactine **F** diactinal polyactine.

Shape massively encrusting (Fig. 11A) with irregular conulose-villose surface. Size of specimen 4 × 3 cm in lateral expansion, 2 cm in thickness. Color (alcohol) red brown.

Skeleton: columnar, with thick short styles at the center of a mass of polyactines, with long thin styles protruding from this skeleton surrounded by shorter centrotylote styles.

Spicules: long thin styles, short thin centrotylote styles, short thick styles, polyactines.

Long thin styles (Figs 11B, B1), relative straight and robust, frequently with subterminal tyle 860–*1041*–1290 × 6–*7.8*–10 µm (De Laubenfels gives: up to 1560 × 12 µm).

Short thin styles (Figs 11C, C1), curved, centrotylote, often with mucronate slightly rugose pointed end, 191–*242.8*–306 × 1.5–*2.4*–3 µm.

Short thick styles (Fig. 11D), smooth, curved evenly, occasionally oxeote, 270–*408.2*–468 × 14–*16.8*–24 µm.

Polyactines (Figs 11E, 11E1, 11F) robust, largely smooth with cladi spined only at the apices (Fig. 11E1), or all cladi smooth. The three- or four claded forms vary widely in size and are sometimes reminiscent of *Trikentrion* spicules. Three-claded forms tend to have longer and thicker lateral cladi than the rare four-claded forms. Basal cladi in three-claded spicules are 33–*48.8*–63 × 8–*11.7*–14 µm, lateral cladi 72–*95.7*–132 × 7–12 µm, while four-claded forms have basal cladi 40–*55.0*–69 × 6–*7.7*–9 µm and lateral cladi 30–*45.1*–57 × 5–*6.3*–7 µm. There are very common diactinal polyactines (Fig. 11F), mimicking oxeas, but recognizable as reduced polyactines by centrotylote swellings and finely spined apices, size 123–*158.3*–202 × 7–*10.2*–14 µm.

##### Distribution.

Southern Californian Bight (San Pedro, Santa Catalina island, La Jolla).

##### Ecology.

On hard substrate, at depths 0–36 m.

##### Discussion.

*Cyamon neon* is unusual among *Cyamon* species by it possession of polyactines with smooth or barely spined cladi, the shape of many of the polyactines mimicking those of *Trikentrion*, and the occurrence of diactinal polyactines. The latter spicules are shared with *Cyamon argon*, which in most respects is similar to *Cyamon neon*. For a comparison between the two species see below in the remarks to *Cyamon argon*. The only other *Cyamon* species in the area is *Cyamon koltuni*, which differs substantially in the bulbous endings of the cladi of the polyactines and absence of the short thin styles.

#### Cyamon argon

Dickinson, 1945

http://species-id.net/wiki/Cyamon_argon

**Figure 12.**
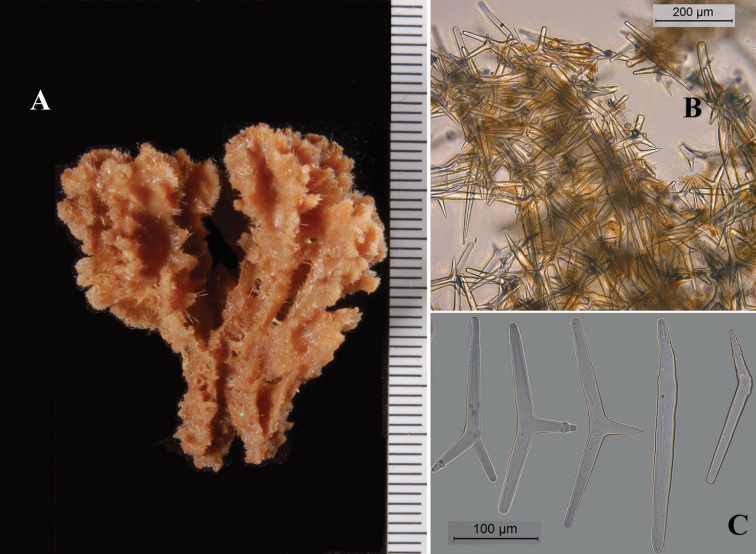
*Cyamon argon* Dickinson, 1945, holotype AHF-NHMLA L35535 (D34), **A** shape (scale mm) (photo Phyllis Sun) **B** microphoto of cross section of skeleton showing columns of styles supported by polyactines **C** microphoto of a range of polyactine shapes.

**Figure 13.**
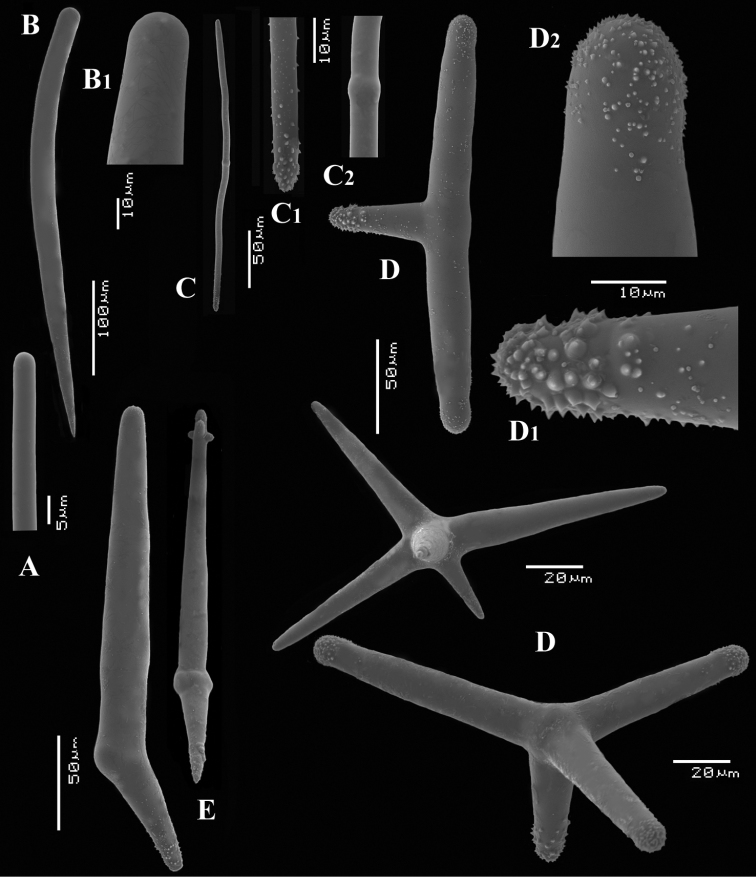
*Cyamon argon* Dickinson, 1945, holotype AHF-NHMLA L35535 (D34), **A** detail of head of long thin style **B** short thick style **B1** detail of head of short thick style **C** short thin centrotylote (strongylo-)style **C1** detail of spined apex of short thin (strongylo-)style **C2** detail of centrotylote part of short thin (strongylo-)style **D** polyactines **D1** heavily spined basal cladus of polyactine **D2** lightly spined lateral cladus of polyactine **E** diactine polyactines.

Figs 12A–C , Figs 13A–G

Cyamon argon Dickinson 1945: 15, pl. 19 Figs 37–38 (Mexican Pacific).

##### Material examined.

Holotype of *Cyamon argon*, AHF-NHMLA L35535, D34, preserved in alcohol, Mexico, Cedros Island, South Bay, Hancock Pacific Expeditions, Velero Station 287–34, 28.09°N, 115.3°W, 18–27 m, among kelp, 10 March 1934.

##### Description.

Shape upright, bilobed thick branches (Fig. 12A), spreading out upwards, with longitudinal grooves and covered in rounded spiny projections and conules. Height and diameter 3.5 cm, stalk approximately 1.5 cm. Colour (preserved) red-brown. Consistency tough, barely incompressible.

Skeleton: axial-columnar, with surface projections formed by the outwardly directed columns (Fig. 12B) branching off from the axial region. Columns have a core of short thick styles and polyactines crowned at the surface by long thin styles accompanied by (rare) short thin centrotylote styles.

Spicules: long thin styles, short thin styles, short thick styles, polyactines.

Long thin styles (Fig. 13A), mostly broken in the slides, one complete one measured 960 × 15 µm.

Short thin centrotylote (Figs 13C, C1, C2), wavy to somewhat crooked, with one end rounded and the other mucronate-spined, 210–*250.6*–348 × 3–*3.6*–4 µm.

Short thick styles (Figs 13B, B1), smooth curved evenly, 350–*480.5*–593 × 15–*32.3*–42 µm.

Polyactines (Figs 12C, 13D-E) two-, three-, four- and five-claded, quite variable in shape and size. T-shaped spicules (Fig. 13D) similar to those found in *Trikentrion* are common. Basal cladi usually prominently spined (Fig. 13D1), lateral cladi finely spined (Fig. 13D2). No entirely smooth spicules were observed. Diactinal spicules (Fig. 13E) with swollen excentrical swellings and spined apices, often sharply angulated. Three-claded spicules with basal cladi 45–*60.7*–78 × 6–*14.9*–22 µm, lateral cladi 30–*110.7*–162 × 5–*17.0*–21 µm. Four-claded spicules have basal cladi 33–*44.8*–51 × 9–*14.9*–21 µm, lateral cladi 63–*86.2*–123 × 7–18 µm. Diactinal spicules: 204–*245.1*–312 × 18–*22.8*–31 µm.

##### Distribution.

Pacific coast of North Mexico.

##### Ecology.

In kelp forest, 18–27 m.

##### Discussion.

As pointed out above, this species is close to *Cyamon neon*, and if more data on variation would become available, it is possible, in view of the nearness of both type localities that the two might be part of a single variable species. The following characteristics are similar between the two: long thin styles of 1000+ µm in length, the possession of short thin centrotylote styles with spined pointed apex (shared with *Cyamon vickersii*), smooth evenly curved short thick styles of 400-500 µm in length, polyactines consisting predominantly of three-claded polyactines with all cladi smooth except for the apices, short basal cladus compared to long lateral cladi, and the frequent occurrence of diactinal polyactines. However, there are also clear differences, which presently preclude synonymization of the two: shape bush-like in *Cyamon argon*, massively encrusting in *Cyamon neon*, thickness of short thick styles in *Cyamon argon* twice that of *Cyamon neon*, basal cladi of the polyactines distinctly spined in *Cyamon argon* whereas these are only rugose or even smooth in *Cyamon neon*, and finally the size (length but also thickness) of the lateral cladi in three-claded polyactines which are usually well over 200 µm long and 20 µm thick in *Cyamon argon*, whereas those of *Cyamon neon* are on average around 150 × 10 µm.

With *Cyamon vickersii*, this species shares a more elaborate, upright growth form, which is otherwise rare in the genus.

#### Cyamon quinqueradiatum

(Carter, 1880)

http://species-id.net/wiki/Cyamon_quinqueradiatum

**Figure 14.**
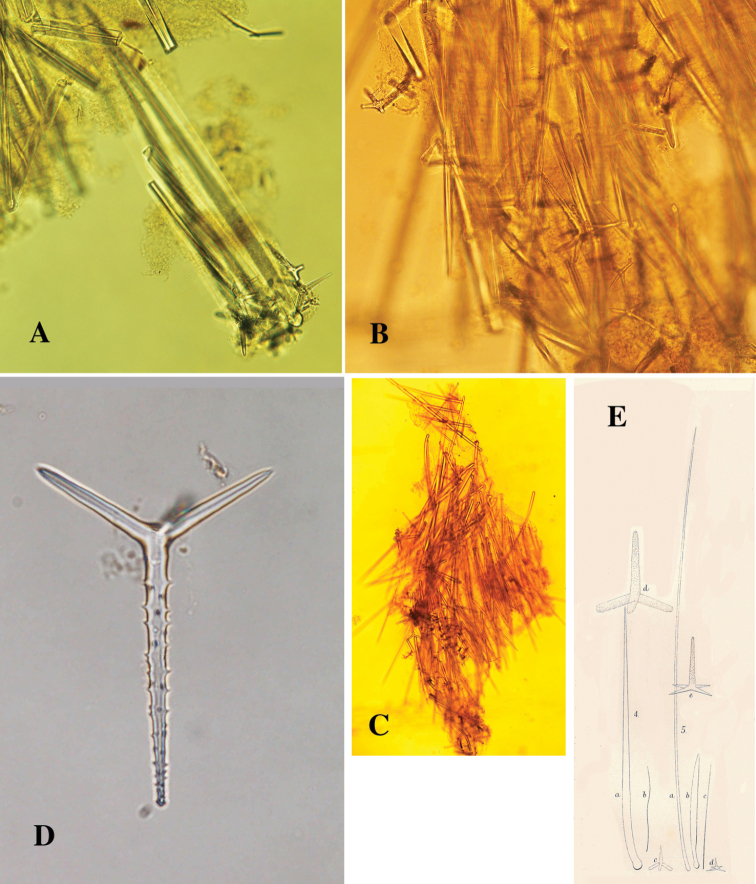
*Cyamon quinqueradiatum* Carter, 1880, images of Dendy’s (1905) non-type slides BMNH 1954.2.23.8 (**A–E**) and *Cyamon quadriradiatum* Carter, 1880 (E left), **A–C** various perpendicular sections showing long subtylostyles and basal polyactines of *Cyamon quinqueradiatum* **D** polyactine of *Cyamon quinqueradiatum* showing spined basal cladus and smooth lateral cladi **E** *Cyamon quadriradiatum* Carter, 1880 and *Cyamon quinqueradiatum* Carter, 1880, original drawings from Carter, 1880, E (right) *Cyamon quinqueradiatum*, right side of figure, showing long subtylostyle, short subtylostyle, thin style, and polyactine with spined basal cladus and smooth lateral cladi E(left) *Cyamon quadriratiatum*, left side of figure, showing long thick style, thin wavy spicule, and entirely spined polyactines.

Figs 14A–D, 14E (right)

Microciona quinqueradiata Carter 1880: 43, pl. IV fig. 5a-e (Gulf of Manaar, India).
Cyamon quinqueradiatum ; Dendy 1905: 178 (Gulf of Manaar, Sri Lanka).

##### Material examined.

Seven slides from the collections of the Natural History Museum, BMNH 1954.2.23.8, made of Dendy’s (1905) topotypical material.

Carter’s specimen from the Gulf of Manaar is apparently lost from the collections of the National Museums Liverpool (Dr Ian Wallace, *in litteris*), no original slides have been found in the Natural History Museum (Ms Emma Sherlock, *in litteris*).

##### Description. 

(Partly from Carter, 1880 and Dendy, 1905). Thinly encrusting, hispid, yellowish brown (alcohol) to cream color (dry). Dendy’s specimen was 1.1 cm in lateral expansion, 3 mm thick. Texture soft.

Skeleton (Figs 14A–C): bundles of subtylostyles and styles standing erect on the substratum, in the basal layer supported by polyactine spicules.

Spicules: predominant spicules are longer and shorter subtylostyles with a minority of thin styles and polyactines.

Subtylostyles, presumably a mixture of undifferentiated long thin styles and short thick styles, with prominent heads, usually lightly and gradually curved, in a large size range, which makes determining an average size meaningless: 129–1989 × 3–33 µm.

Thin styles, tapering gradually to thinly pointed curved ends, size range limited, 492–698 × 3–5 µm. Dendy believed these spicules to be growth stages of the subtylostyles, but we regard them, like Carter, as a separate spicule category.

Polyactines [Figs 14D, 14E(right)], predominantly five-claded (a few four-claded forms were observed), with the basal cladus relatively finely spined, the lateral cladi smooth, with mucronate, occasionally bifid ends, basal cladi 45–*62.8*–93 × 4–*5.9*–11 µm, lateral cladi 31–*38.4*–51 × 3–*4.8*–7 µm.

##### Distribution.

Only known from the Gulf of Manaar.

##### Ecology.

Deep water (not specified).

##### Discussion.

As pointed out above, Mauritanian *Cyamon arguinense* sp. n. shares many features with Indian Ocean *Cyamon quinqueradiatum*, including the smooth lateral cladi and the lack of differentiation of the long thin and short thick styles. Although the *Cyamon* nature of this species has never been challenged, it is nevertheless obvious from the original description and drawing by Carter (1880) and the subsequent record of Dendy (1905) that the polyactines of this species have only their basal cladi spined, an alleged prominent and discriminating feature of the genus *Trikentrion*. We have confirmed single cladus spination by examining a series of slides of Dendy’s material. The structure of the skeleton with longer and shorter styles originating from a basal mass of polyactines is characteristic for *Cyamon*. This indicates that emphasis on a single spined cladus versus all cladi spined as a difference between *Cyamon* and *Trikentrion* is wrong. See further discussion below. Among the species of *Cyamon* the present species also stands out by the extreme length variation of the structural subtylostyles, assuming these are homologous with the ‘short thick styles’ of many other *Cyamon* species, and perhaps related to it, the absence of a category of long thin styles. The thin styles observed above are assumed by their size to be homologous to the peripheral short thin styles surrounding the long thin styles in other species.

#### Cyamon quadriradiatum

(Carter, 1880)

http://species-id.net/wiki/Cyamon_quadriradiatum

Fig. 14E (left)

Microciona quadriradiata Carter 1880: 42, pl. 4 fig. 4 (Gulf of Manaar, India).

##### Material examined.

None. Type material apparently lost from the collections of the National Museums Liverpool (Dr Ian Wallace, *in litteris*), no slides have been found in the Natural History Museum (Ms Emma Sherlock, *in litteris*).

##### Description.

(From Carter, 1880). Thinly encrusting, hispid, color when dry dark brown. Spicules (Fig. 14E, left) of three kinds, long thick styles with a globular tyle, size given as 1042 × 41 µm, short thin ‘crooked’ styles, length 347 µm, and robust four-claded polyactines with all cladi entirely spined, length of cladus given as 76 µm.

##### Distribution.

Gulf of Manaar, Southeastern India.

##### Ecology.

No data.

##### Discussion.

This species needs redescription, but the long thick styles in combination with the densely spinous polyactines appear sufficiently distinct. Nevertheless there is a resemblance to the polyactines of *Cyamon aruense*, see above.

#### Cyamon hamatum sp. n.

urn:lsid:zoobank.org:act:BA36E82A-F8CB-4FA4-B589-DDE8694C220D

http://species-id.net/wiki/Cyamon_hamatum

Figs 15A–C

Cyamon vickersii ; Burton and Rao 1932: 355 (S India).
Not: Cyamon vickersii (Bowerbank, 1864)

##### Material examined.

**Type specimen**: Holotype (schizotype), 1 slide BMNH 1931.1.1.19a, labeled *Cyamon vickersii (Bow.) Ind. Mus. Coll*. in Burton’s handwriting. Presumably the type specimen was at one time lodged in the collections of the Indian Museum, Kolkata, India, but present whereabouts are unknown. It is likely housed in the Zoological Survey of India, Kolkata.

**Type locality**: India, 21 miles S.W.W. of Mangalore, 4 May 1888.

##### Description.

partly from Burton and Rao 1931:

*The single representative is a portion of a dull brown spherical mass. It agrees with the specimen described by Dendy (l.c.) except that the longest ray of the pseudactines bears a few recurved rays on the shaft and a crown of spines at the apex; the basal rays of these spicules have spines at the apex only; and the styli are very scarce. Locality. – 21 miles S.W.W. of Mangalore, S India (4^th^ May 1888)*.

**Figure 15.**
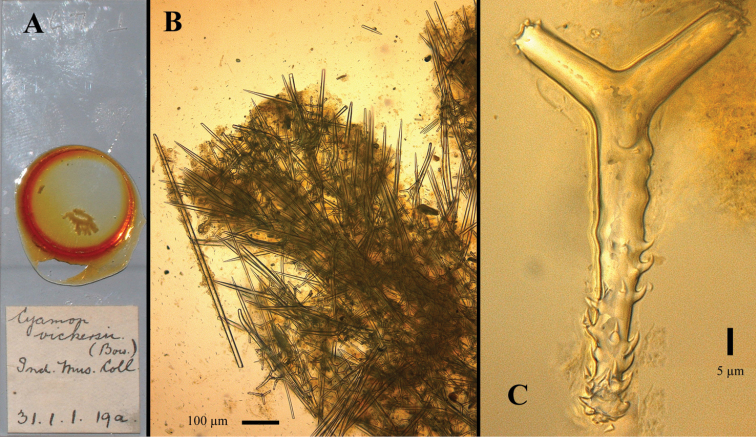
*Cyamon hamatum* sp. n. (*Cyamon vickersii* sensu Burton and Rao 1932), from S India, **A** slide BMNH 1931.1.1.19a, labeled ‘*Cyamon vickersii* (Bow.) Ind. Mus. Coll.’ in Burton’s handwriting **B** microphoto of section of skeleton made from the slide **C** characteristic polyactine with recurved hook-like spines on basal cladus and short stubby lateral cladi.

The slide (Fig. 15A) contains thick sections of the skeleton, showing the usual columnar structure of thick styles and polyactines (Fig. 15B). The slide allows the recognition and measurement of the spicule complement.

Spicules: long thin styles, short thin centrotylote styles, short thick styles, polyactines.

Long thin styles, not frequent, invariably broken, longest fragment measured 1300 × 30 µm.

Short thin styles, wavy outline, faintly centrotylote, under light microscopy mostly looking smooth but occasionally some spines are visible on the pointed end and also in at least one spicule two spines on the rounded end, 272–*313.2*–355 × 2.5–*3.4*–5 µm

Short thick styles, smooth, curved rather strongly near the rounded end: 421–*495.6*–604 × 16–*19.9*–31 µm.

Polyactines (Fig. 15C), predominantly three-claded, but occasionally four-claded, with long basal clades with prominent recurved hook-like spines and with short, stubby lateral cladi spined only at the bluntly rounded apices, basal cladi 104–*114.2*–126 × 11–*14.8*–21 µm, lateral cladi 42–*47.8*–65 × 10–*11.7*–20 µm.

##### Etymology.

The adjective *hamatus* (L.), means *provided with hooks*.

##### Distribution.

South India.

##### Ecology.

No data.

##### Discussion.

It is with some hesitation that we decided to name this scanty material as a valid new species. Although measurements of the megascleres conform to or are close to those of *Cyamon vickersii*, the shape and spination of the polyactines is distinctly different, as Burton & Rao already observed. With their strong hooks on the basal cladi and the peculiar short *crowned* lateral cladi the polyactines are different from any other known *Cyamon*.

#### Cyamon (?) spinispinosum

(Topsent, 1904)

Figs 16A–E

Hymeraphia spinispinosa Topsent 1904: 162, pl. 14 fig. 9 (Azores).
Acantheurypon spinispinosum ; Topsent 1928: 293 (Azores).
Acantheurypon incipiens Topsent 1928: 293, pl. 10 fig. 10 (Azores).
Cyamon spinispinosum ; Stephens 1921: 61 (Ireland); Van Soest et al. 2007: 130 (Rockall Bank, W of Ireland).

##### Material examined.

Twenty six samples in the ZMA Porifera collection, preserved in alcohol, all from Rockall Bank, approximately 55.4N, 15.8W, depth 500–900 m, collected during MOUNDFORCE 2004 and BIOSYS 2005 cruises with RV *Pelagia*. Type material:Monaco Oceanographic Museum, not examined.

##### Description.

**Figure 16.**
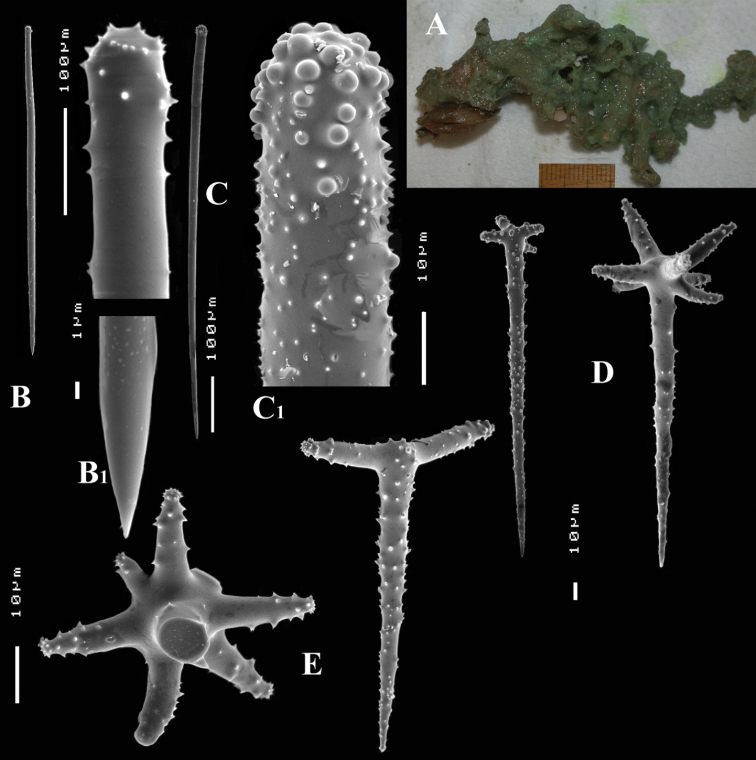
*Cyamon spinispinosum* (Topsent, 1904), ZMA Por. 19422, from SE Rockall Bank, North Atlantic **A** shape encrusting deep sea coral (scale = 1 cm) **B** short style, lightly spined at the head **B1** details of apices of short style **C** long style, coarsely spined **C1** detail of head of long style **D** various shapes of polyactines **E** detail of the cladome of a seven-claded polyactine.

Pale greenish encrustations (Fig. 16A) on deep-sea coral branches, surface irregularly conulose-hispid. Consistency soft. Dimensions up to 15 × 6 cm in widest expansions, thickness approximately 1 mm.

Skeleton: basal mass of polyactines, usually a single layer of spicules, with basal cladi pointing outwards and lateral cladi spread out on the substrate, taking up the position of echinating acanthostyles as in *Hymedesmia* or *Clathria (Microciona)*. Single long styles with heads embedded in the layer of polyactines, surrounded by groups of short styles.

Spicules: long styles, short styles, polyactines.

Long styles (Figs 16C, C1) with upper parts heavily spined, becoming gradually smooth toward the pointed end, only a few were found to be complete, 657–*737.2*–822 × 32–*35.5*–38 µm.

Short styles (Figs 16B, B1), very abundant, heads slightly spined, shaft smooth, faintly polytylote, pointed end tends to be slightly mucronate, 302–*324.1*–366 × 7–*8.4*–10 µm.

Polyactines (Figs 16D-E), with 3–8 cladi, usually with a long and prominent basal cladus and short irregular lateral cladi (Fig. 16E), heavily spined without smooth areas, basal cladi 90–*151.3*–234 × 9–*11.2*–14 µm, lateral cladi 15–*27.3*–36 × 6–*7.4*–12 µm.

##### Distribution.

Azores, Ireland, also Norway (P. Cárdenas, pers. comm.). A common North Atlantic bathyal species (van Soest et al. 2007 report 110 specimens collected on Rockall Bank, W of Ireland).

##### Habitat.

Encrusting deep-sea corals at depths from 500–900 m.

##### Discussion.

This is a deviating *Cyamon* with several unique features not shared by the majority of the species. Both monaxone megascleres are partially heavily spined, and the raspailiid feature of a long thin style surrounded by short thin styles is absent. The polyactines resemble echinating acanthostyles by their long basal cladus and crown of short irregular lateral cladi. These spicules may be assumed to bridge the gap between the polyactines with more or less equal length cladi and acanthostyles with heavily knobbed and spined heads such as found in some myxilline genera (*Hymedesmia* Bowerbank, 1864, *Discorhabdella* Dendy, 1924) and in the raspailiid genus *Eurypon* Gray, 1867. Additionally, it occurs in cold deep-sea habitats unlike all other *Cyamon* species. It is likely that this species does not belong in *Cyamon*, but we will await additional (molecular) evidence before removing it from the genus

#### Genus Trikentrion

Ehlers, 1870

http://species-id.net/wiki/Trikentrion

##### Type species:

*Spongia muricata* Pallas, 1766 (by monotypy).

##### Definition

**(emended).** Cyamoninae with reticulate skeleton containing polyactine spicules of which the basal cladi are provided with hook-like spines in mature condition, and if present choanosomal oxeas. Microscleres trichodragmas. Additional longer and shorter thin styles are usually present in peripheral regions.

##### Remarks.

Polyactine spicules are genuinely polyaxone, with axial canals visible in all of the predominantly three, occasionally four- or two cladi. As will be demonstrated below, none of the specimens of the type species we were able to examine, including the neotype, possess the raspailiid synapomorphy of peripheral long styles surrounded by short styles, despite Hooper’s (2002) description of the type species where such spicules were mentioned. Possibly, but unlikely, these spicules are present in living condition, because we only had dry old specimens available and the peripheral skeleton may have become abraded. It seems likely that Hooper’s (2002) description was based on a contaminated spicule slide. All other *Trikentrion* species do have the long and short styles as a peripheral skeletal feature, and in that sense the type species appears a deviating representative of the genus.

*Trikentrion* differs from *Cyamon* in its possession of choanosomal oxeas (whereas *Cyamon* has styles), but several species, *Trikentrion catalina*, *Trikentrion helium* Dickinson, 1945 and *Trikentrion africanum* sp. n., are lacking these spicules. The polyactines of *Trikentrion* differ from those of *Cyamon* in having only the basal clade provided with strong hook-like spines, with the lateral cladi smooth; also the shape is often Y- or T-shaped. As demonstrated above, these differences are not entirely exclusive, because *Cyamon arguinense* sp. n. and *Trikentrion quinqueradiatum* also have only the basal cladus spined, whereas Y- and T-shaped polyactines occur in *Cyamon neon* and *Cyamon argon*. Finally, all species of *Trikentrion* described below have abundant trichodragmas, which are entirely lacking in *Cyamon* species.

### Description of the type species of *Trikentrion*

#### Trikentrion muricatum

(Pallas, 1766)

http://species-id.net/wiki/Trikentrion_muricatum

**Figure 17.**
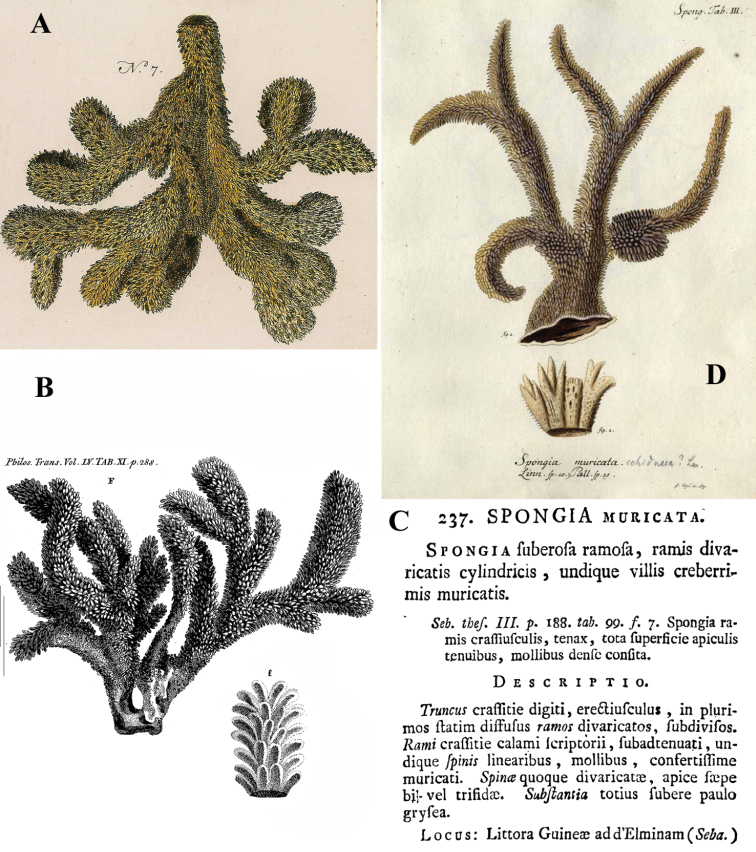
*Trikentrion muricatum* (Pallas, 1766), early illustrations and original descriptions **A** Seba’s drawing (1734-1765, volume 3 pl. 99 fig. 7) of *Spongia muricata* as indicated by Pallas (1766) **B** Ellis’s (1766) drawing of a branched tuberculated sponge from W coast of Africa **C** original description of *Spongia muricata* by Pallas (1766) **D** Esper’s (1794) drawing of *Spongia* muricata from W coast of Africa. See text for further explanation.

**Figure 18.**
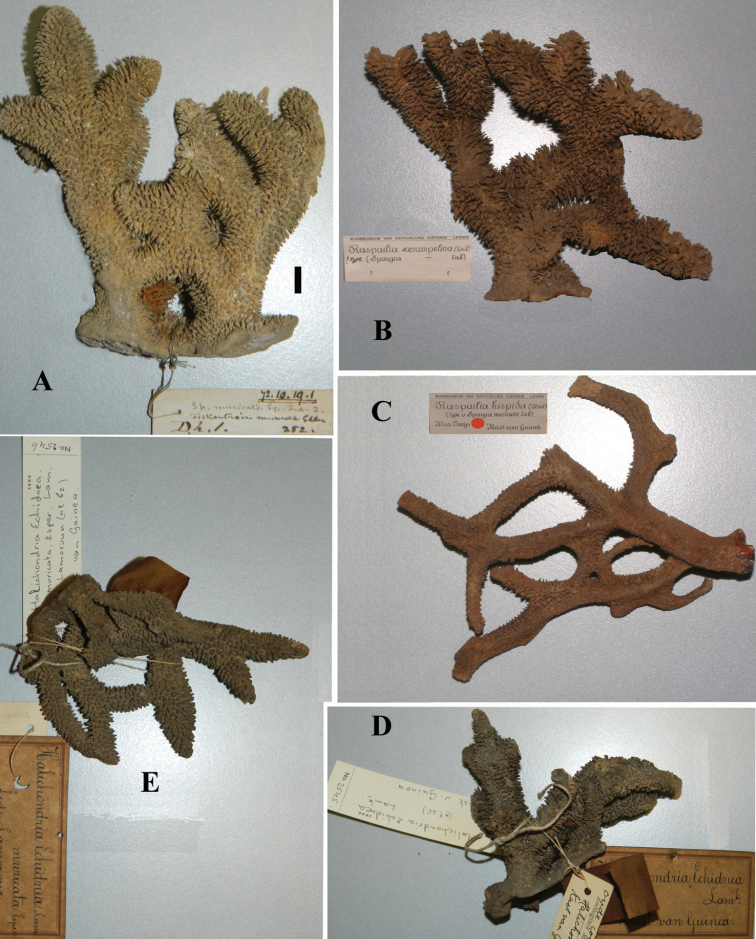
*Trikentrion muricatum* (Pallas, 1766), early museum specimens **A** BMNH 1872.10.19.1 redescribed by Carter (1879) designated neotype herein **B** RMNH Por. 309 labeled *Spongia xerampelina* Lamarck, showing strong resemblance to Seba’s (1734-1765) drawing but lacking sufficient data **C** RMNH Por. 306 labeled *Spongia muricata* Lamarck showing strong resemblance to Esper’s (1794) drawing but lacking sufficient data **D** ZMA Por 02545 labeled *Spongia echidnea* resembling Esper’s (1794) drawing but lacking sufficient data **E** ZMA Por 02546 labeled *Spongia echidnea* resembling Seba’s (1734-1765) drawing but lacking sufficient data. See text for further explanation.

**Figure 19.**
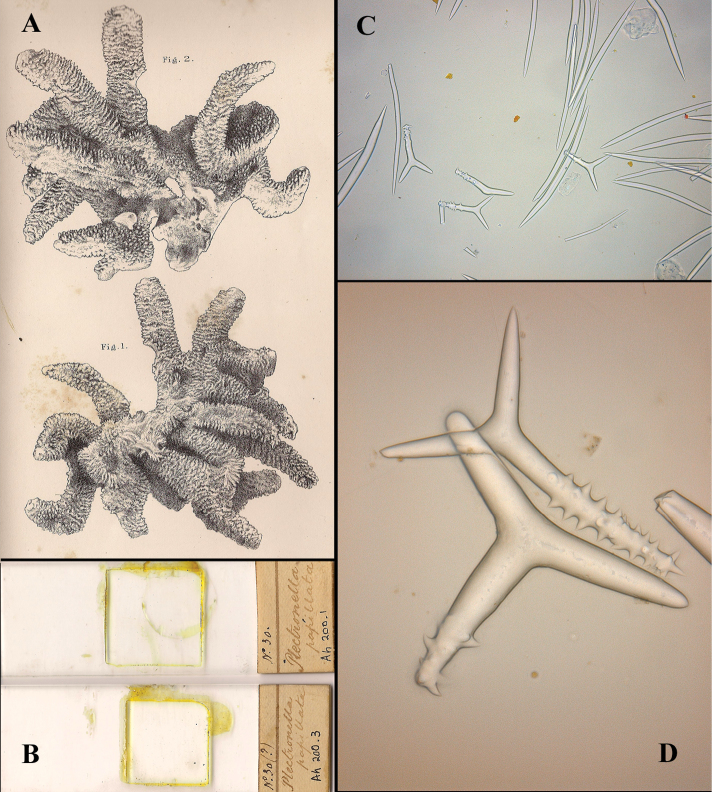
*Plectronella papillosum* Sollas (1879), assumed to be a junior synonym of *Trikentrion muricatum* (Pallas, 1766), **A** shape, repinted from Sollas (1879: pl. 4) **B** remaining type material in the form of two microscopic slides BMAG Ah 200.1, 200.3 **C** overview of spicules present in one of the microscopic slides **D** polyactine spicules present in one of the microscopic slides.

**Figure 20.**
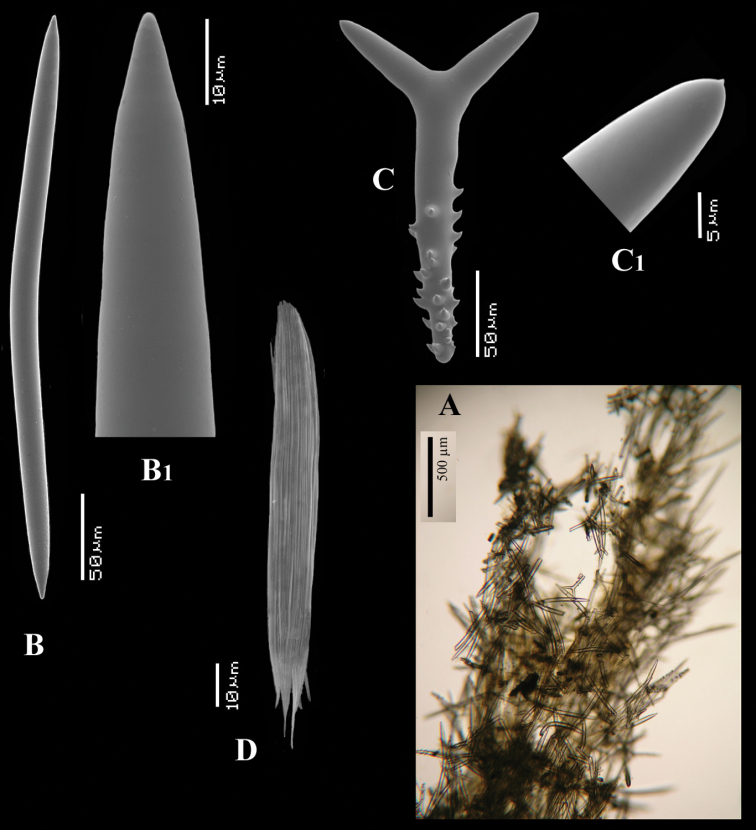
*Trikentrion muricatum* (Pallas, 1766), neotype BMNH 1872.10.19.1 **A** microphoto of cross section of peripheral skeleton **B** oxea **B1** detail of one of the apices of an oxea **C** polyactine **C1** detail of apex of lateral clade **D** trichodragma.

Figs 17A–D , 18A–E , 19A–D , 20A–D

Spongia muricata Pallas 1766: 389 (referring to Seba 1734–65, volume III pl. 99 fig. 7, Ghana); Esper 1794: 185, pl. 3 (Ghana).
(not: Linnaeus 1759: 1348; 1767: 1298 = unrecognizable; nec: Lamarck 1814: 448 = *Raspailia hispida*, cf. Topsent 1932: 107).
Unnamed branched tuberculated sponge; Ellis, 1766: pl. 11 fig. F (West Africa).
? Spongia echidnea Lamarck, 1814: 448 (West Africa).
Trikentrion muricatum ; Ehlers 1870: 6; Carter 1879: 293, pl. 27 fig. 13 (Ghana); Burton 1956: 133, 142 (Ghana); Hooper 2002: 499, figs 18A-G.
Plectronella papillosa Sollas 1879: 17, pls 4-5.
? Ptilocaulis echidnaeus ; Topsent 1932: 108, pl. III fig. 3.

##### Remarks.

The identity of the sponge named *Spongia muricata* by Pallas, which is assumed to be the type species of *Trikentrion*, is not straightforward. The first use of the name combination stems from Linnaeus (1759: 1348), who described it as:

*S. ramosissima, poris cylindricis subulatis prominentibus aequalibus multifidis hispidis*, without further indication of where it had been collected or by whom. The Latin name *muricata* is generally considered to mean spined (after the name of a mollusk (*Murex*) yielding a purple dye, cf. Brown 1985), for sponges a hardly distinguishing feature. The description speaks of cylindrical pores, which is quite vague, and this character does not occur in any specimen discussed in this paragraph and below. Pallas (1766: 389), employed the name combination also, but indicated and described the sponge figured in Seba’s (1734–1765) volume 3 pl. 99 fig. 7 as representing his *Spongia muricata*. Pallas did not refer to Linnaeus’ name, nor did his description remind in any aspect of Linnaeus’ description. Seba’s figure is here reproduced in Fig. 17A, and Pallas’s description in Fig. 17C. Pallas also quoted Elmina on the coast of Guinea (now Ghana) as the locality of the specimen based on Seba’s information. In the same year (or perhaps one year before), Ellis (1765–1766), pictured a similar sponge (*a branched tuberculated sponge* here reproduced in Fig. 17B), stating that it originated *from the Cape Coast Castle in Africa* (which could very well be the same locality Elmina), but not naming it. In his 1767 edition, Linnaeus again described *Spongia muricata*, replacing the first word of the 1759 edition, *Spongia ramosissima* by the text *S. foraminulata ramosissima angulata tenax*, followed by the same words as previously (reaffirming the unrecognizable shape of the sponge). He also added that it originated from *O. Aethiopico* (Indian Ocean). He now gave three sources for his record of this species, viz. Mus. Tessin 118, plate II figure 1, Seba’s volume 3 plate 99 fig. 7, and Pallas’ record. Finally, Gmelin in Linnaeus 1788: 3821, admits that the species is from *Guineae littorea*, quoting a.o. Pallas (1766) and Linnaeus (1767), but remarkably omitted any reference to Linnaeus (1759). Linnaeus’ and Pallas’ (Seba’s) specimens of *Spongia muricata* have never been identified in later collections (but see below), and their identity remains a matter of speculation. In 1794, Esper extensively described *Spongia muricata* and his figure is here reproduced in Fig. 17D. This time, the specimen, stated to be from *Guinea*, from cliffs near Elmina (= Ghana), was still extant in the collections of the University of Erlangen (Germany) in 1870, when Ehlers revised some of Esper’s specimens (Ehlers, 1870). He detected the triactine spicules and erected the genus *Trikentrion* for it. His description included some measurements of the spicules: oxeas 354–414 × 16 µm, and polyactines, with basal cladi 95 µm and lateral cladi 72 µm, which data conform closely with those presented below for the species. However, since Ehlers’ redescription, the whereabouts of the Esper material is unknown and it must be assumed lost. Lamarck (1814: 448) misinterpreted *Spongia muricata* and his material was assigned to *Raspailia hispida* (Montagu, 1818) by Topsent (1932: 107). Possibly, *Spongia echidnea* Lamarck, 1814 is a junior synonym of *Spongia muricata* Pallas, because the redescription and figured specimen of Topsent (1932: 108, as *Ptilocaulis echidnaeus*) reminds rather strongly of it. However, Topsent fails to mention the presence of polyactine spicules.

To conclude: the identity of *Spongia muricata* is not unequivocal, primarily due to the unrecognizable description of Linnaeus (1759) and the likelihood that he used the name for an unknown species from the Indian Ocean. Pallas’ description in combination with Seba’s figure make it likely that his *Spongia muricata* indeed is what we now know as *Trikentrion muricatum*, but uncertainty reigns due to the fact that only Esper’s, not Pallas’, material was shown to possess the synapomorphy of the polyactine spicules. It appears highly necessary to fix *Spongia muricata* as a *Trikentrion*, by assigning a neotype. In the absence of any topotypical fresh material of the species we are forced to choose dry old collection material.

A likely candidate is the assumed *type* of *Trikentrion muricata* housed in the Natural History Museum, London, BMNH 1872.10.19.1 (see Fig. 18A), with *schizotype* ZMB 7160, on the basis of which Carter (1879) redescribed and illustrated the species *Trikentrion muricatum*, and which subsequently formed the basis of the Systema Porifera entry of the genus and its type species. This is not likely to be Seba’s specimen, nor Esper’s because the locality data (though from Ghana as well) do not indicate Elmina. In addition to this specimen, the Natural History Museum collections incorporate a *schizotype* of *Spongia muricata*, *Coast of Guinea*, BMNH 1954.2.20.93, which appears unimportant for the present choice of neotype because it is not a *Trikentrion*, but an unidentified species of *Axinella* Schmidt, 1862.

The choice of a neotype again is complicated due to a recent discovery in the collections of the Naturalis Biodiversity Center at Leiden (NBC) of four *old collection* specimens, RMNH Por. 306 and 309, and ZMA Por. 02545 and 02546, which are sufficiently similar to Seba’s and Esper’s plates to raise the suspicion that they could belong to one of the original specimens of *Spongia muricata*.

RMNH Por. 309 (see Fig. 18B) is labeled *Raspailia xerampelina (Lmk) ? type (Spongia --- Lmk)* without further information, and this specimen bears an overall strong likeness to Seba’s plate. RMNH Por. 306 (see Fig. 18C) is labeled *Raspailia hispida (Mont.) type van Spongia muricata Lmk, Mus. Parijs, Kust van Guinée* (translation: type of Spongia muricata Lamarck, from the Paris Museum, Coast of Guinea). If the specimen is compared to the plate of *Spongia muricata* of Esper one is compelled by the overall likeness of the two (though it is not an exact likeness). ZMA Por. 02545 (see Fig. 18D) is labeled *Halichondria echidnaea Lmk no. 55 Kust van Guinea*, ZMA Por. 02546 (see Fig. 18E) is labeled *Halichondria echidnaea Lam / muricata Esper fide Lamouroux no. 62 Kust van Guinea*. Both ZMA specimens bear some resemblance to Seba’s and Esper’s plates.

The skeleton and spicules of all five specimens conform with the descriptions of Ehlers (1870) and Carter (1879).

The reason for the names on the labels of the specimens of the NBC and the referral to the Paris Museum is explained in Holthuis (1995): during the French occupation in 1795 of the Republic of Holland in the Napoleontic period, Dutch collections were confiscated and relocated to the Paris Museum. Some time after the end of the emperorship of Napoleon in 1815, negotiations between The Netherlands and France resulted in a *donation* of specimens, notably duplicates from Lamarck’s collection, to the then founded Rijksmuseum of Natural History at Leiden. Dozens of sponge specimens labeled with Lamarck’s names are incorporated in the RMNH collections, but because the redescription of Lamarck’s sponges by Topsent (1931, 1932, 1933) was initiated after the transfer of specimens to Leiden, there is often little correspondence between the identities of the MNHN and RMNH specimens bearing labels with the same original Lamarck names. Topsent (1932) identified Lamarck’s *Spongia muricata* as *Raspailia hispida*, and this was duly taken over by past curators of the Leiden specimens, who apparently were unaware of the discrepancies between the Paris and Leiden specimens. It is possible, that the Lamarck specimen redescribed in Topsent (1932 as *Raspailia hispida*), is not Lamarck’s original specimen, because this may have ended up in the Leiden or Amsterdam collections.

In view of the uncertain history of the NCB specimens and the more precise data available for the Natural History Museum, London specimen, we here designate BMNH 1872.10.19.1 as the neotype of *Spongia muricata*, the type species of the genus *Trikentrion*.

It is a pleasure to be able to announce that material of *Plectronella papillosa* Sollas, 1879, since long known to be a junior synonym of *Trikentrion muricatum* through its excellent description by Sollas, but otherwise never redescribed, has been discovered in the collection of the Bristol Museum and Art Gallery, in the form of 2 slides labeled *No. 30 Ah.200.1, 200.3* (see Fig. 19B), containing cross sections of the skeleton and dissoluted spicules. We can confirm that *Plectronella papillosa* is a junior synonym and that details in the slides conform closely to those of *Trikentrion muricatum* (see Fig. 19C–D).

##### Material examined.

Neotype (designation herein): BMNH 1872.10.19.1 from Volta River, Fantee, Ghana, *presented by Gov. Ussher*. Schizotype ZMB 7160 of the same;

RMNH Por. 306, *Spongia muricata Lamarck, Coast of Guinea*;

RMNH Por. 309, *Spongia xerampelina* *Lamarck*, no further data;

ZMA Por. 02545, 02546, *Halichondria echidnaea / muricata Lamarck, coast of Guinea*;

BMAG Ah 200.1, 200.3, 2 slides labeled *Plectronella papillosa no. 30*, no further data.

##### Description.

Wide basal holdfast upon which are erected groups of cylindrical branches, more or less in one plane, each branch usually with one or two dichotomous secondary branches, often also with anastomosing branches. Size of neotype (Fig. 18A) 13.5 × 12 × 5 cm of the whole group of branches, diameter of individual branches 1-1.5 cm. Sollas’ specimens (Fig. 19A as *Plectronella papillosa*) were described as being 20 × 20 cm, with branch diameter 2-3 cm. The other specimens are similar in size, but slightly smaller. Surface densely covered with broad, laterally flattened papillae, 1-4 mm in size (reminding of the surface projections of *Ptilocaulis* Carter, 1883). In some specimens the papillae are partially abraded (e.g. RMNH Por. 306, see Fig. 18C) giving the sponge a less striking aspect. Consistency (dry) hard, incompressible, crumbly. No live color has been reported in the literature, but color plates of Seba (Fig. 17A) and Esper (Fig. 17D) show a light orange brown color.

Skeleton (Fig. 20A): predominantly a wide-meshed reticulation of tracts of robust smooth oxeas, with little axial and extra-axial specialization. The polyactines are common in peripheral regions. No longer or smaller peripheral styles have been found in any of the examined specimens.

Spicules: Oxeas, polyactines, trichodragmas.

Choanosomal ‘true’ oxeas (Figs 19C, 20B, B1, not to be confused with diactinal polyactines), fat, fusiform, tapering gradually to sharp points, overall size (of all specimens examined) 222–*376.2*–528 × 13–*19.9*–31 µm, in the neotype: 287–*351.5*–432 × 13–*17.2*–26 µm.

Polyactines (Figs 19D, 20C), predominantly three-claded Y-shaped, rarely T-shaped, occasionally diactinal, with prominent hook-like spines on the basal clade (undeveloped spicules with smooth basal clade), and mucronate or nipple-like endings on many of the lateral cladi; overall size of basal clade (of all specimens examined) 78–*111.7*–156 × 12–*19.2*–27 µm, lateral cladi 42–*67.2*–84 × 12–*16.7*–27 µm, of neotype: basal clade 78–*100.2*–118 × 12–*19.4*–25 µm, lateral cladi 58–*69.2*–84 × 13–*16.3*–21 µm.

Trichodragmas (Fig. 20D), straight or sinuous, overall size (of all specimens examined) 57–*82.0*–102 × 4–*9.7*–18 µm, of neotype: 63–*87.8*–102 × 9–*12.8*–18.

##### Distribution.

Tropical West Africa. Type from ‘Elmina, Guinea’ (Pallas, 1766; Esper, 1794), now situated in Ghana. Further specimens were reported mostly from Ghana (neotype (Carter, 1879): Volta; Burton, 1956: *Gold Coast*), or locality was unknown (Sollas, 1879), or more general (*coast of Guinea*).

##### Ecology.

Depth range: no definite data, but probably shallow water, growing on rocks.

##### Discussion. 

The species must have been of common occurrence off the coast of Ghana in 18^th^ century as there are a fair lot of specimens available from that age and region in several natural history museums. Curiously, no fresh material is known to exist, so the species remains ill-known. *Trikentrion muricatum* differs substantially from all other *Trikentrion* species described below in the lack of peripheral styles. Further differences are robust oxeas, up to twice as long and thick as those of the two other oxea-bearing species (*Trikentrion laeve* Carter, 1879 and *Trikentrion flabelliforme*), while the three remaining species (*Trikentrion helium*, *Trikentrion catalina* and *Trikentrion africanum* sp. n.) lack the oxeas entirely.

### Descriptions of further species assigned to *Trikentrion*

#### Trikentrion laeve

Carter, 1879

http://species-id.net/wiki/Trikentrion_laeve

Figs 21A–F

Trikentrion laeve Carter 1879: 294, pl. 27 figs 9-12 (West Africa);
(Not: Carter 1882: 294 = *Trikentrion flabelliforme*; nec: Burton 1948 = *Trikentrion africanum* sp. n.)

##### Material examined.

Holotype: BMNH 1848.10.4.6 (additional numbers Dh.2, 252), West Africa, coll. Rev. Allen; label text, presumably by Carter, reads *Trikentrion Ehlers, very long acuates*.

##### Description.

**Figure 21.**
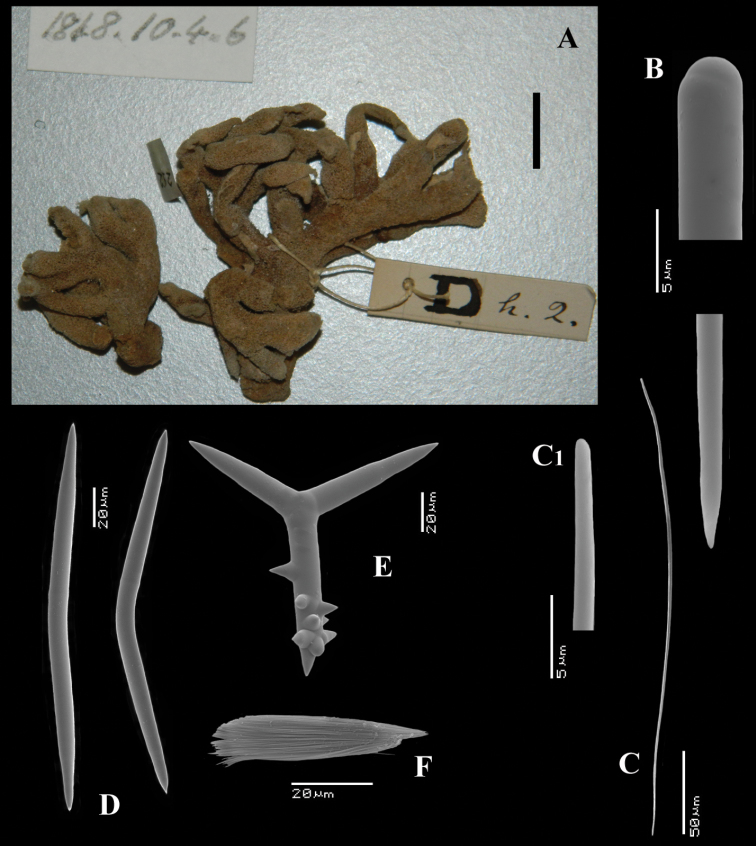
*Trikentrion laeve* Carter, 1879, holotype BMNH 1848.10.4.6, **A** shape of holotype specimen (size bar = 1 cm) **B** details of apices of long thin style **C** short thin style **C1** detail of rounded head of short thin style **D** oxeas **E** polyactine **F** trichodragma.

Multi-branched bush (Fig. 21A), with single stalk of 1.5 cm high, 0.8 cm diameter, from which cylindrical branches spread out dichotomously, ending in approximately 26 smaller terminally rounded branches. Size of entire specimen, which is broken in two unequal parts, 4.5 × 5.5 × 3 cm. Surface optically smooth, but microhispid, with punctate appearance. Consistency (dry) crumbly compressible, colour beige-purplish.

Skeleton: a comparatively loose reticulation of oxeas echinated sparingly with polyactines, forming rounded or squarish meshes of 150–200 µm diameter, with 5 or more oxeas to the sides, no axial specialization. Peripherally there are numerous long thin styles, accompanied by short thin styles.

Spicules: long thin styles, short thin styles, oxeas, polyactines, trichodragmas.

Long thin styles (Fig. 21B), rather curved, 750–*921.8*–1062 × 4–6.6–9 µm.

Short thin styles (Figs 21C, C1), often modified to thin oxeotes, wispy, curved, 234–*312.9*–433 × 0.5–*1.4*–2.5 µm.

Choanosomal ‘true’ oxeas (Figs 21D) (not to be confused with diactinal polyactines), straight, or more often centrotylote or abruptly curved, with pointed ends, very common, 175–*204.1*–242 × 6–*9.6*–13 µm.

Polyactines (Fig. 21E), usually three-claded, occasionally four-claded or diactinal, mostly Y-shaped, less often equiangular, with the basal ray provided with strong hook-like spines, basal cladi 59–*69.6*–89 × 10–*11.9*–15 µm, lateral cladi 47–*63.4*–75 × 9–*10.7*–13 µm.

Trichodragmas (Fig. 21F), straight or curved, 32–*48.2*–60 × 4–*8.2*–11 µm.

##### Distribution.

*West Africa* (Carter, 1879).

##### Ecology. 

Probably from shallow water or washed up on the beach. No further data.

##### Discussion.

This is the first redescription after Carter’s report, which is accurate but deficient in omitting the trichodragmas and short thin styles. This is also the first depiction of habit of the specimen and with the details provided here the species is now at least properly described, but it remains ill known. Carter (1879) differentiated this species from *Trikentrion muricatum* by emphasizing the presence of ectosomal long styles, which appear lacking in *Trikentrion muricatum*. The shape and surface characteristics of the two are also quite different, and sizes of the oxeas and polyactines are considerably smaller than in *Trikentrion muricatum*.

Carter (1882) reported this species from Australia, but from his description it is clear that it concerns the species later described as *Trikentrion flabelliforme* Hentschel, 1912. The two differ significantly in shape (T.* flabelliforme* being thinner or thicker bladed, lacking rounded branches forming a three-dimensional bush). The three other species of *Trikentrion* differ by lacking oxeas.

Burton (1948) reported this species from the République du Congo, more to the south, but this specimen lacks oxeas and has a different shape. It is assigned to a new species (*Trikentrion africanum* sp. n.) below.

#### Trikentrion flabelliforme

Hentschel, 1912

http://species-id.net/wiki/Trikentrion_flabelliforme

Figs 22A–D , 23A–E

Trikentrion laeve sensu Carter 1882: 294 (West Australia) (not: Carter 1879)
Trikentrion flabelliforme Hentschel 1912: 373, pl. 13 fig. 9, pl. 20 fig. 32 (Aru Islands, Indonesia); Capon et al. 1986: 6545; Hooper 1991: 1298, Figs 61–62, 109h-I (North and West Australia); Hooper 2002: Figs 18H–J.

##### Material examined.

Holotype missing from SMF, but a paralectotype fragment is present in the Natural History Museum, BMNH 1931.8.4.57, which was examined by JH in 2000, type locality: Indonesia, Aru Islands, 4–15 m depth.

ZMA Por. 02426, preserved in alcohol, Siboga Exped. Stat. 273, Aru Islands, Indonesia, pearl banks off Pulau Jedan, 5.4134°S, 134.6677°E, depth 13 m, 23–12–1899.

RMNH Por. 978, preserved in alcohol, Siboga Exped. Stat. 273, same data;

ZMA Por. 14022 and 14023, preserved in alcohol, East Point, Darwin, Northern Territories, Australia, 10 m, 29–11–1987, coll. J.N.A. Hooper nrs 8 and 9;

ZMA Por. 16049, dry old collection material without data.

##### Description.

**Figure 22.**
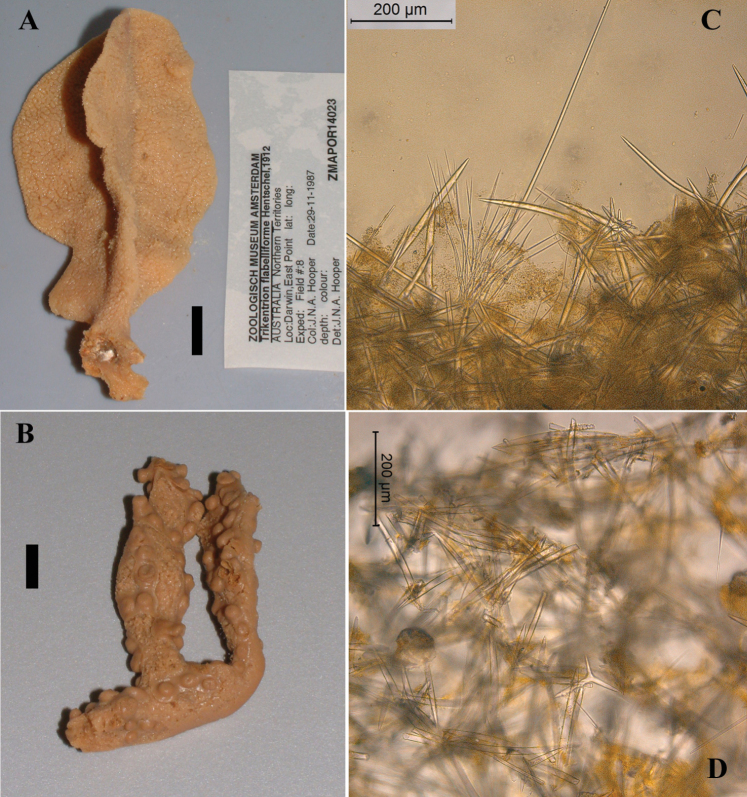
*Trikentrion flabelliforme* Hentschel, 1912, **A** flabellate specimen ZMA Por. 14023 from Darwin, North Australia (scale bar = 1 cm) **B** branching-digitate specimen RMNH Por. 978 infested with zoanthids from Aru Islands Indonesia (scale bar = 1 cm) **C** peripheral skeleton of ZMA Por. 14023 showing raspailiid character of long thin style sheathed in a bouquet of short thin styles **D** thick section of choanosomal skeleton of ZMA Por. 14023.

Two distinct shapes, flabelliform (Fig. 22A), 6–26 × 4–19 cm high and wide, 0.2–1.4 cm thick) and digitate (Fig. 22B), up to 15 cm high, with flattened branches of up to 1.5 cm thickness (summary of many specimens described in Hooper, 1991). Flabelliform specimens may have blades at right angles (see Fig 22A). Frequently, the digitate specimens are infested with zoanthids (Fig. 22B). Surface optically smooth, microhispid, with characteristic pattern of fine meandering grooves. Texture firm. Colour orange-red, blood-red (shallow water) to beige (deep water).

Skeleton (Figs 22C–D): reticulated, square meshed or polyangular (Fig. 22D), with loose extra-axial and spongin-rich axial spicule tracts cored by oxeas, echinated by triactine polyactines; at the surface protruding long thin styles are surrounded by bouquets of short thin styles (Fig. 22C).

Spicules (Figs 23): Long thin styles, short thin styles, oxeas, polyactines, trichodragmas.

Long thin styles (Fig. 23A), curved, slim, 405–*870.3*–1034 × 3–*7.3*–9µm.

Short thin styles (Fig. 23B), thinly fusiform, 182–334.7–392 × 0.5–*1.8*–4 µm.

Choanosomal genuine oxeas (Figs 23C, C1), not to be confused with diactinal polyactines, evenly or more angularly curved, apices mucronate and many have minute spines visible under SEM (Fig. 23C1), sizes 135–*287.7*–340 × 5–*16.8*–22 µm.

Polyactines (Figs 23D, D1), rare in some specimens, predominantly three-claded, with prominent spines on the basal ray, and minute apical spines on the lateral rays (Fig. 23D1) visible only under SEM, occasionally strongly curved diactines or – often smaller – tetractines, basal cladi 96–*109.5*–123 × 10–*13.1*–17 µm, lateral cladi 51–*70.0*–84 × 9–*12.6*–17 µm.

Trichodragmas (Figs 23E, E1) with individual raphides showing rugose surface (Fig. 23E1), sizes 35–*59.6*–88 × 6–*8.6*–12 µm.

**Figure:**
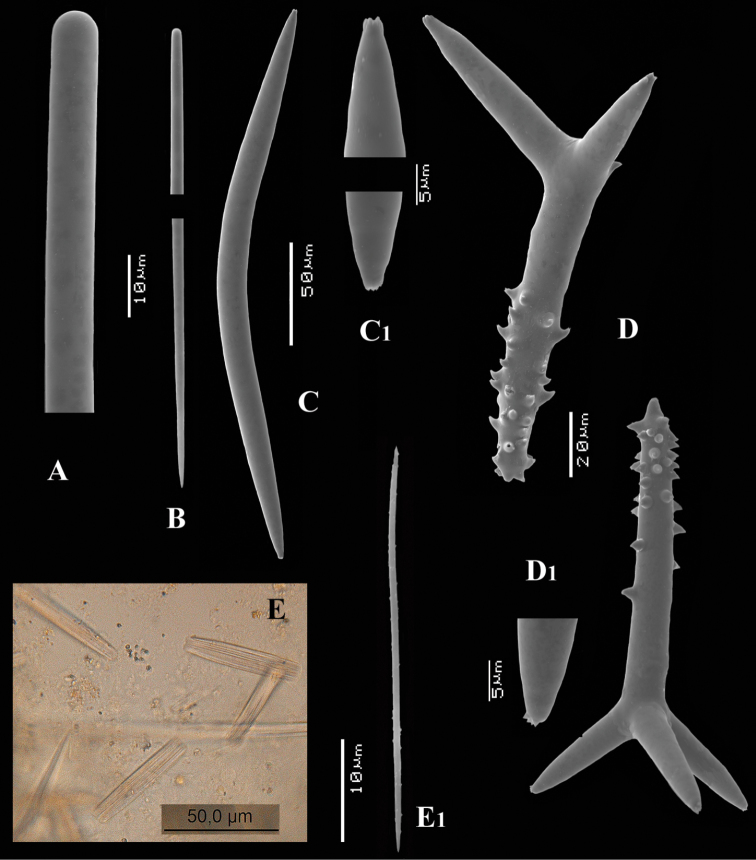
**Figure 23.** *Trikentrion flabelliforme* Hentschel, 1912, spicules of ZMA Por. 14023, **A** detail of rounded end of long thin style **B** details of short thin style **C** oxea **C1** details of apices of oxeas showing minute spines **D** three- and four claded polyactines **D1** detail of apex of lateral clade of polyactine showing minute spines **E** microphoto of trichodragmas **E1** individual raphide dissociated trichodragma showing rugosities.

##### Distribution.

Arafura Sea, N and W Australia.

##### Ecology.

Shallow subtidal to offshore deeper water.

##### Discussion.

The species was erroneously attributed to Carter 1882: 294, allegedly as *Trikentrion laeve* var.* flabelliforme*, by Hooper (1991). This is a manuscript name because Carter did not name his Australian flabellate specimen, merely referring it to *Trikentrion laeve*. That species is West African and described above as a distinct new species.

The two ‘growth forms’ are rather distinct, but distribution, skeleton, and spicules are similar and overlapping enitirely, making it impossible to separate the forms further. The digitate form is often overgrown with a zoanthid species, both in Australian (Hooper, 1991) and Indonesian (RMNH Por. 978) specimens. The shape of *Trikentrion flabelliforme* reminds of Californian *Trikentrion catalina* and *Trikentrion helium*, but spiculation in these species differs substantially by their lack of proper choanosomal oxeas. Comparative variation in shape is also recorded for *Trikentrion helium* (see below).

The apices of the oxeas and the polyactines show minute spines, which is here interpreted as a unique feature. It violates the rule that in *Trikentrion* only the basal, not the lateral cladi of the polyactines have spines, but there is little correspondence with the lateral cladus spination in *Cyamon*.

This is the only *Trikentrion* species that appears to be widespread and common. Chemistry of *Trikentrion flabelliforme* includes unique indoles (Capon et al. 1986).

We studied an Indonesian specimen from the ZMA collection labeled *Trikentrion elegans* Lendenfeld identified by Burton (ZMA Por. 02402, Siboga Exped. Stat. 303, Timor, Samau Island, Haingsisi, 10.2050S, 123.4591E, 23 m), which has the shape and skeletal structure of a small digitate *Trikentrion flabelliforme*, including ectosomal long thin styles (up to 1350 × 12 µm), short thin styles (300–400 × 1–3 µm), a choanosomal reticulation of robust oxeas (300–400 × 15–20 µm) and large amounts of trichodragmas (60–110 × 5–15 µm), but lacking polyactine spicules entirely. In view of the occasional rarity of these spicules observed in some specimens of *Trikentrion flabelliforme*, it is likely that it is a ‘deficient’ specimen of this species. Anecdotal records of *Trikentrion flabelliforme* from northern Australia have also occasionally encountered similarly deficient specimens (B. Alvarez, pers.comm.). The locality of the Siboga specimen is neatly inbetween the type locality and the North and West Australian localities.

#### Trikentrion helium

Dickinson, 1945

http://species-id.net/wiki/Trikentrion_helium

Figs 24A–E

Trikentrion helium Dickinson 1945: 15, pl. 20 figs 39–40 (Mexican Pacific); Luke 1998: 10 (La Jolla, Southern California).
? Trikentrion catalina ; Gómez et al. 2002: 230, fig. 5 (Mexican Pacific).

##### Material examined.

Holotype AHF-NMHLA L-35535 (D33), preserved in alcohol, Hancock Pacific Expeditions, Mexican Pacific, Cedros island, South Bay, approximately 28.07°N, 115.3°W, 18–27 m depth, Velero Station 287–34, 10 March 1934.

**Figure 24.**
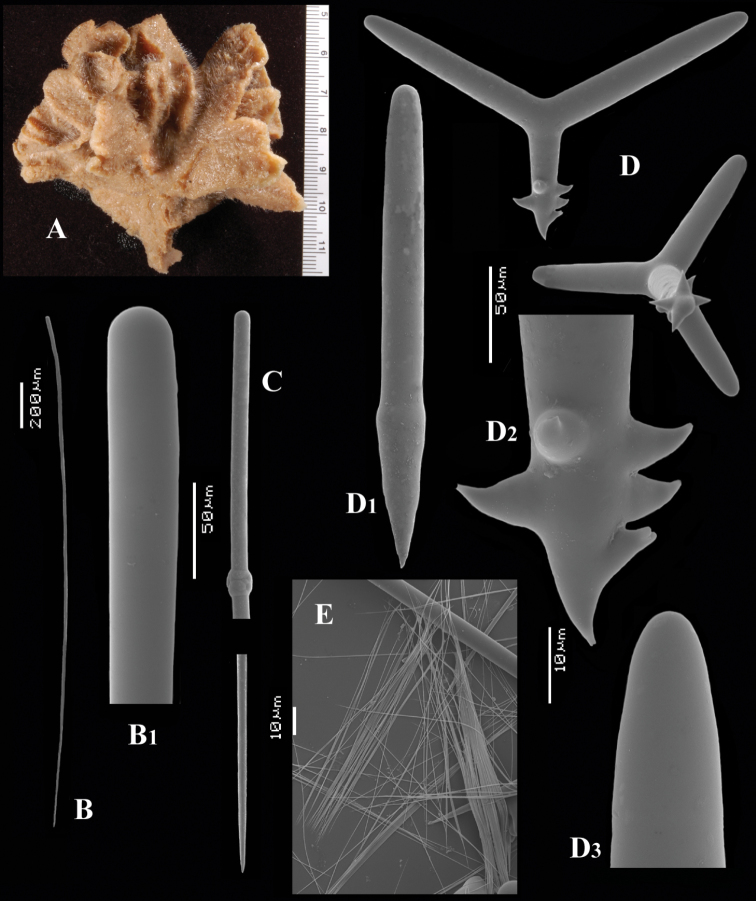
*Trikentrion helium* Dickinson, 1945, holotype AHF-NMHLA L-35535 (D33), **A** shape of holotype (photo Phyllis Sun) **B** long thin style **B1** detail of head of long thin style **C** details of short thin style **D** polyactines **D1** reduced diactinal polyactine **D2** detail of spination of basal clade of three-claded polyactine **D3** detail of apex of lateral clade of three-claded polyactine **E** trichodragmas.

**Description.** Undulating thin-bladed sheets together forming a bushy mass (Fig. 24A) of 7 × 5 × 5 cm. The surface bears a thick spicule brush of 3 mm thickness. Conistency firm, brittle. Colour reddish brown (alcohol).

Skeleton: built chiefly by polyactines (no oxeas), supporting the bases of long styles, which are surrounded by dense brushes of short thin styles.

Spicules: long thin styles, short thin styles, polyactines among which numerous diactinal forms, trichodragmas.

Long thin styles (Figs 24B, B1), variably thinner and thicker, but not divisible in two thickness categories, 952–*1808.1*–3393 × 18–*25.8*–42 µm.

Short thin styles (Fig. 24C), usually curved, and often with a subterminal tyle, 372–*438.0*–510 × 2.5–*3.1*–3.5 µm.

Polyactines (Fig. 24D), predominantly wide-angled triactines (Fig. 24D), with basal cladi provided with course conical spines (Fig. 24D2), lateral cladi usually much longer than basal cladi, with smooth, rounded endings (Fig. 24D3); basal cladi 66–*105.4*–144 × 8–*22.1*–30 µm, lateral cladi 96–*146.5*–192 × 7–*23.6*–36 µm; few, mostly smaller, tetractinal polyactines occur, with cladi 27–63 × 9 µm; more frequently diactinal reduced polyactines (Fig. 24D1) occur, asymmetrical, sometimes style-like, smooth, recognizable by an excentric swollen tyle, 192–*235.2*–306 × 13–*19.8*–27 µm.

Trichodragmas (Fig. 24E) abundant, occurring throughout the choanosomal and ectosomal regions, 84–*100.7*–123 × 10–*12.1*–15 µm. Individual raphides less than 0.5 µm in thickness.

##### Distribution.

The holotype was collected in the Southern Californian Bight (Mexican Pacific). Luke (1998) records several specimens from La Jolla, California (USA). If specimens of Gómez et al. (2002) belong to this species, it occurs in the Sea of Cortez and further south along the Mexican Pacific coast.

##### Ecology.

Rocks and reefs at depths of 15–28 m.

##### Discussion.

The trichodragmas were not cited in the original description. *Trikentrion helium* shares the dominance of three-claded polyactines with relatively long lateral cladi with *Trikentrion catalina* (see below), to which it seems closely related. This species differs quite strongly from the other *Trikentrion* species by its possession of numerous diactinal or style-like reduced polyactines, which resemble, but clearly are not proper, oxeas like those of *Trikentrion muricatum* and *Trikentrion flabelliforme*. The spicules are recognizable as polyactines by the substantial difference between the smoothly rounded end, resembling the ends of the lateral cladi of the three-claded polyactines, and the dissimilar pointed end which shows an irregular surface and is connected to the other end by a swollen, often irregular middle part. Their lengths coincide with the added lengths of a lateral and a basal clade of the three-claded forms. Such reduced diactinal polyactines are also common in *Cyamon neon*.

The specimens described by Gómez et al. (2002) under the name *Trikentrion catalina* were branching erect rather than bladed, but branches were typically flattened, 2–4 mm in thickness. We reassign these specimens to *Trikentrion helium*, because they apparently possess oxea-like polyactines [described as oxeas but confirmed as reduced polyactines by one of us (JLC)], whereas in *Trikentrion catalina* there are neither oxeas forming the main skeleton as in *Trikentrion flabelliforme* nor diactinal polyactines as in *Trikentrion helium*. The difference in shape between the type of *Trikentrion helium* and Gómez et al.’s specimens is here considered to be mere variation (comparable to variation in *Trikentrion flabelliforme*, see above) but further studies might reveal there is more specific diversity along the Pacific coast of Mexico.

#### Trikentrion catalina

(Sim & Bakus, 1986)

http://species-id.net/wiki/Trikentrion_catalina

Figs 25A–F

Cyamon catalina Sim and Bakus 1986: 18, fig. 4; Lee et al. 2007: 211 (California).
Not: Trikentrion catalina ; Gómez et al. 2002 = *Trikentrion helium*.

##### Material examined.

Holotype USNM 33631, preserved in alcohol, California, Santa Catalina Island, Bird Rock, 33.45°N, 118.4833°W, on rocky cliff at 50 m depth, coll. K. McCleneghan.

Not examined: paratype BMNH 1985 (reg. nr. unknown), Santa Catalina Island, Ship Rock, on rock at 46 m depth, coll. R. Given.

##### Description.

**Figure 25.**
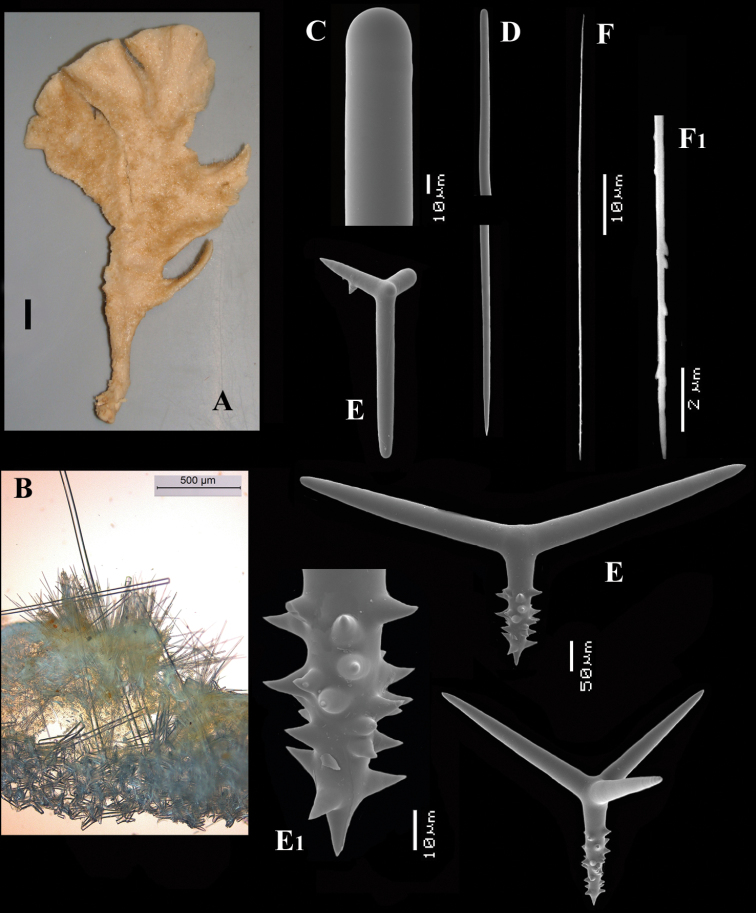
*Trikentrion catalina* (Sim & Bakus, 1986), holotype USNM 33631, **A** shape of holotype specimen (scale bar = 1 cm) **B** cross section of skeleton **C** detail of head of long thin style **D** details of short thin style **E** three- and four-claded polyactines **E1** detail of spined basal clade of polyactine **F** raphide **F1** detail of raphide showing spination.

Flabelliform sponge (Fig. 25A), measuring 15 × 8 by 0.4 cm, attached to rocks by a 3 × 0.6 cm stalk. Surface hispid. No oscules apparent. Consistency firm and leathery. Color reddish orange (alive), pale beige (alcohol).

Skeleton (Fig. 25B): choanosome densely packed with three-claded polyactines; ectosome with long, relatively thick styles surrounded by dense bouquets of short thin styles; trichodragmata commonly observed especially in the peripheral parts.

Spicules: long (thin) styles, short thin styles, polyactines, trichodragmas.

Long (thin) styles (Fig. 25C), usually broken and only a few could be measured: 1400–5400 × 8–40 µm, so not really thin.

Short thin styles (Fig. 25D), 130–*611.3*–730 × 3–*5.6*–8 µm,

Polyactines (Figs 25E, E1), predominantly three-claded, with spined shorter basal cladi (Fig. 25E1), occasionally with few or no spines on the basal cladi, and smooth, longer, relatively pointed lateral cladi; occasionally four-claded; size basal cladi 78–*98.7*–126 × 16–*25.3*–31 µm, lateral cladi 156–*197.7*–236 × 18–*24.4*–29 µm.

Trichodragmas: straight, with lightly spined raphides (Figs 25F, F1), 63–*79.3*–88 × 7–*10.2*–13 µm.

##### Distribution.

Santa Catalina Island, Southern California.

##### Ecology.

On rocks, from 46–50 m depth.

##### Discussion. 

This species is assigned to *Trikentrion*, because of the flabellate shape resembling *Trikentrion flabelliforme* Hentschel (1912), the sagittal polyactines, three-claded with spines only on the basal cladus, and the possession of trichodragmas. It is similar to *Trikentrion helium* in the lack of genuine choanosomal oxeas, and the short basal cladi of the polyactines. Remarkably, when describing *Trikentrion catalina*, Sim & Bakus (1986) did not notice - they did not discuss *Trikentrion helium* - the similarities with their species. Specimens assigned to *Trikentrion catalina* by Gómez et al. (2002) are considered to be long to *Trikentrion helium* (see above).

#### Trikentrion africanum sp. n.

urn:lsid:zoobank.org:act:0807BE5A-BD22-4C6A-907D-3772E69CA479

http://species-id.net/wiki/Trikentrion_africanum

Figs 26A–E

Trikentrion laeve ; Burton 1948: 757 (Congo); Burton 1956: 142.
Not: Trikentrion laeve Carter 1879.

##### Material examined.

**Type specimen**: Holotype BMNH 1939.2.20.9, preserved in alcohol.

**Type locality**: République du Congo, Pointe Noire, approximately at 4.7667°S, 11.8333°E, coll. E. Darteville, June 1938.

##### Description.

**Figure 26.**
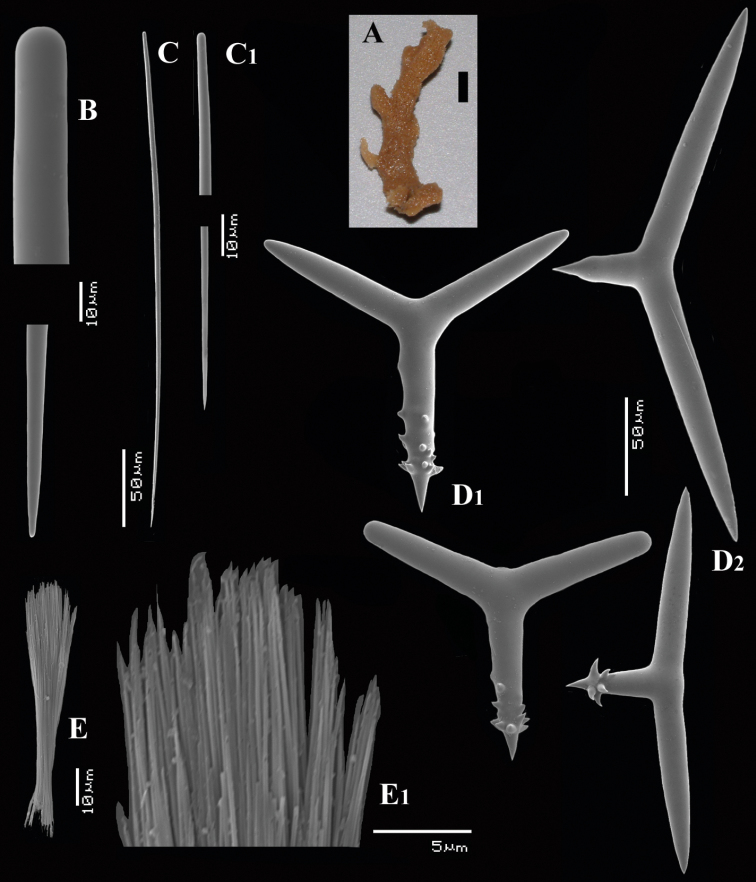
*Trikentrion africanum* sp. n., holotype BMNH 1939.2.20.9, **A** shape of holotype (scale bar = 1 cm) **B** details of long thin style **C** short thin style **C1** details of short thin style **D** various shapes of polyactines **E** trichodragma **E1** detail of trichodragma.

Upright flattened branch with two or three short side projections (Fig. 26A), with wider base and a cut-off upper ending, possibly the specimen is only a fragment as base and apex look damaged. Length of holotype 6.5 cm, diameter 1.5 cm at the base, 1 cm higher up. Side projections only on one side of the branch, less than 1 cm long and 0.5 cm thick, with rounded apex. Surface uneven, somewhat hispid. No apparent oscules. Consistency firm. Colour (alcohol) red-brown.

Skeleton: a dense mass of polyactines, towards the periphery surrounding long thin styles and short thin styles, which are embedded in the skeleton more so than in other *Trikentrion* species. No oxeas present, but T-shaped polyactines with very short basal clade appear to have taken the position of oxeas.

Spicules: long thin styles, short thin styles, polyactines, trichodragmas.

Long thin styles (Fig. 26B), smooth, straight, usually broken, so only a small number (five) were available for length measurements, 295–*870.4*–1394 × 9–*14.6*–24 µm.

Short thin styles (Fig. 26C, C1), straight or gradually curved, 192–*241.1*–358 × 2–*2.3*–3 µm.

Polyactines (Fig. 26D), basically three-claded, with the basal clade provided with strong conical spines near the apex. Two major morphological types appear dominant, those with almost equiangular outline (Fig. 26D1), and T-shaped forms with very short basal clade (Fig. 26D2), which is occasionally entirely smooth; basal cladi 27–*51.3*–96 × 11–*13.7*–21 µm, lateral cladi 33–*96.3*–121 × 9–*13.9*–19 µm.

Trichodragmas (Figs 26E, E1), straight or sometimes curved sinuously, up to 50 or more individual raphides with apical spines, 49–*54.4*–61 × 5–*7.7*–11 µm.

##### Etymology.

The name is anadjective referring to the type locality.

##### Distribution. 

République du Congo.

##### Ecology. 

Shallow water

##### Discussion.

Burton (1948, 1956) assigned this material to the relatively unknown species *Trikentrion laeve* Carter without any morphological information. This is obviously wrong, a.o. because that species has abundant oxea megascleres, lacking in the present material. Carter’s *Trikentrion laeve* was expressly differentiated from *Trikentrion muricatum* in its possession of long thin styles, which are indeed absent in *Trikentrion muricatum*. Both *Trikentrion muricatum* and *Trikentrion laeve* were described and illustrated by Carter to have a strong complement of oxeas (see also above). Their function appears to have been entirely taken over by the polyactine spicules in the present material.

The lack of choanosomal genuine oxeas is shared with Californian *Trikentrion catalina* and *Trikentrion helium*, but these species have flabelliform or bladed shape and much larger polyactine spicules.

### Key to the species of *Cyamon* and *Trikentrion*

Below the species of *Cyamon* and *Trikentrion* considered valid are keyed out. See Table 1 for a summary of recognized species and Table 2 for a summary of their characters.

**Table:** 

1	Trichodragmas absent, polyactines are predominantly four-claded or with more cladi, usually shaped equiangular, choanosomal megascleres if present thick styles	(*Cyamon*) 2
–	Trichodragmas present, polyactines predominantly three-claded Y-shaped, choanosomal megascleres thick oxeas, sometimes absent, but no thick styles	(*Trikentrion*) 13
2	Thicker and thinners styles both heavily spined on the head and more lightly spined along the shaft , polyactines irregular	*Cyamon spinispinosum*
–	All styles smooth, polyactines predominantly regular	3
3	Polyactines in two distinct size categories, the smaller of which is ‘double’	*Cyamon amphipolyactinum* sp. n.
–	No double polyactines	4
4	Polyactines with only the basal cladi spined or rugose	5
–	Polyactines with all cladi spined or rugose	6
5	Thin styles fusiform and centrotylote	*Cyamon arguinense* sp. n.
	Thin styles not centrotylote	*Cyamon quinqueradiatum*
6	Ectosomal short thin styles with rugose or spined pointed end, often also with an angular bend	7
–	Ectosomal thin styles straight, lacking spines or rugose ending or they are entirely absent or not differentiated from long thin styles	10
7	Diactinal polyactines present (differentiated from true oxeas by a rugose or irregular condition of one of the apices)	8
–	No diactinal polyactines	9
8	T-shaped three-claded polyactines common, choanosomal styles averaging 30 µm in thickness, shape a little bush	*Cyamon argon*
–	Polyactines more regular, choanosomal styles averaging 16 µm in thickness, shape a massive encrustation	*Cyamon neon*
9	Polyactines predominantly three-claded, with a long basal cladus with hook-like spines and shorter only terminally spined lateral cladi	*Cyamon hamatum* sp. n.
–	Polyactines predominantly four-claded, with little distinction in length and spination of all cladi	*Cyamon vickersii*
10	Ectosomal thin styles have a faint centrotylote condition, polyactine spicules are heavily and entirely spined	*Cyamon aruense*
–	Ectosomal thin styles present but lacking a centrotylote condition	11
11	Short thick styles absen	*Cyamon quadriradiatum*
–	Short thick styles present	12
12	Polyactine spicules have swollen apices, but these are not developed into prominent knobs	*Cyamon agnani*
–	Polyactine spicules have prominent spined knobs on the lateral cladi	*Cyamon koltuni*
13	Shape rounded branches	14
–	Shape with flattened blades	16
14	Styles absent	*Trikentrion muricatum*
–	Styles present	15
15	Choanosomal genuine oxeas present	*Trikentrion laeve*
–	Oxeas absent	*Trikentrion africanum* sp. n.
16	Choanosomal genuine oxeas present	*Trikentrion flabelliformis*
–	Choanosomal genuine oxeas absent, but diactinal polyactines may be present	17
17	Shape a single large blade, with dense spicule pelt, styles up to 5.5 mm	*Trikentrion catalina*
–	Shape a bladed bush, hispid, but not with a dense pelt, styles up to 3.5 mm	*Trikentrion helium*

### Geographic distribution of species of *Cyamon* and *Trikentrion*

**Figure 27.**
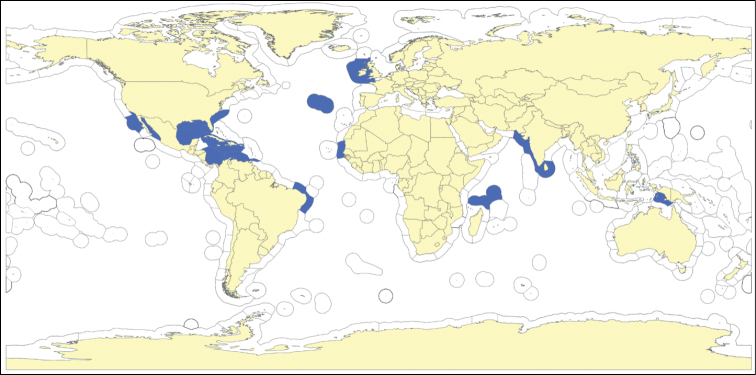
Idealized global distribution of the genus *Cyamon*, showing presence of the genus in Marine Ecoregions of the World (Spalding et al. 2007).

With the new records from Mauritania, South Carolina and the reassigned Brazil record, the genus *Cyamon* appears to have a circumglobal warmer water distribution (Fig. 27), commonly observed in many shallow-water sponges (Van Soest 1994b; Van Soest et al. 2012). Gaps in this distribution appear to be the NW Pacific (Japanese and Chinese waters) and the SW Pacific (Australian and New Zealand waters), and the absence in the Mediterranean is also noteworthy. An odd outlier occurrence is that of *Cyamon spinispinosum* (bathyal North Atlantic), while the concentrated occurrence in the tropical East Atlantic and the warm temperate North East Pacific (three species each) is striking.

**Figure 28.**
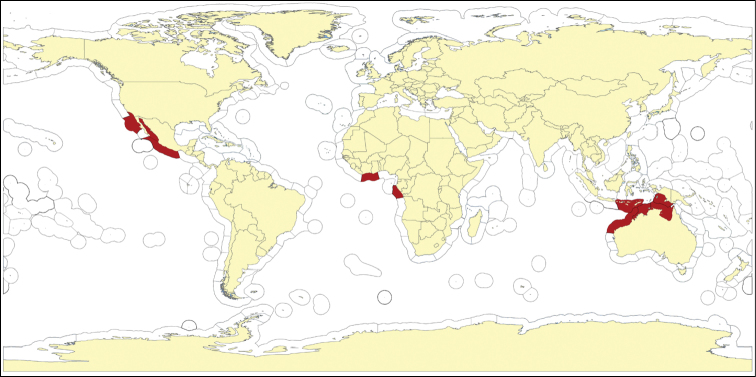
Idealized global distribution of the genus *Trikentrion*, showing presence of the genus in Marine Ecoregions of the World (Spalding et al. 2007).

Species assignable to the genus *Trikentrion* are also found in the warmer waters of all three oceans (Fig. 28), but so far the genus is not recorded from the Central West Atlantic. In contrast, West African waters appear to have a concentrated occurrence of *Trikentrion* species.

It is likely that more species of both genera will be discovered in the near future.

## Discussion

The two genera were independently erected contemporarily (1867 vs 1870), but at first *Cyamon* was ignored (Higgin, 1877; Carter, 1879). Topsent (1889) attempted to synonymize the two on account of the polyactine spicules characterizing both, but he picked the junior name *Trikentrion*. Most subsequent authors kept the two genera as separate and in the latest comprehensive classification of the Porifera (Hooper and Van Soest 2002) this was maintained. Different authors were not consistent in outlining the differences between the two. Mostly, the emphasis was laid on the single vs the overall spination of the cladi of the polyactines. Other features variously indicated as differences, such as growth form (encrusting vs erect), skeleton (plumose vs. reticulate) and choanosomal megascleres (styles vs oxeas) need critical reexamination in recorded specimens. Based on specimens described here as belonging to *Cyamon* species and *Trikentrion* species, we constructed a matrix of characters found in both putative genera (Table 3). From this table it is apparent that four features appear to be more or less consistently different between the two groups. (a) Shape, with the majority of *Cyamon* thickly or thinly encrusting, whereas only in two species the shape is rather more massive or lobate (the type, *Cyamon vickersii* and *Cyamon argon*), with *Trikentrion* never thinly encrusting, always erect and usually elaborate, thick branches or flabellate. In conclusion: this appears to be a fairly consistent difference, although shape is variable and probably not operational for a clear distinction. We do not consider the skeletal structure, plumose in *Cyamon* and reticulate in *Trikentrion*, as an independent character, but assume here that elaborate shape can only be achieved by reticulate organization of the skeleton. (b) The choanosomal megascleres, with *Cyamon* having thick, mostly short, terminally curved styles, and *Trikentrion* thick short oxeas if present (not present in *Trikentrion helium*, *Trikentrion catalina* and *Trikentrion africanum* sp. n.). The thick short oxeas and styles in the two genera could be expressions of the same spicule type, as both are similar in dimensions and tend to be entirely smooth (except *Cyamon spinispinosum*). Nevertheless, the conclusion is that possession of choanosomal ‘true’ oxeas distinguishes *Trikentrion* from *Cyamon*. (c) The presence (*Trikentrion*) or absence (*Cyamon*) of trichodragmas. This appears so far a clear and absolute difference between the two. (d) The spined cladi of the polyactines differ mostly also in spination: coarse recurved spines in *Trikentrion*, finer spined in *Cyamon* (excepting *Cyamon arguinense* sp. n. and *Cyamon hamatum* sp. n.). Most *Cyamon* species have spines on all cladi, whereas *Trikentrion* polyactines have spines only on the basal clade (or are entirely smooth). However, there is no absolute distinction because *Cyamon arguinense* sp. n. and to a lesser extent *Cyamon quinqueradiatum*, both with only spines on the basal cladi, bridge the gap between the polyactines of *Cyamon* and *Trikentrion*. Also, the diactinal polyactines of *Cyamon neon* and *Trikentrion helium* appear quite similar.

**Table 3.** Putative similarities and differences of *Cyamon* and *Trikentrion*

Character	State	*Cyamon*	*Trikentrion*
Shape			
	thinly encrusting	√	–
	lobate	√	√
	branching	√	√
	flabellate	–	√
	hispid surface	√	√
Architecture			
	plumose	√	–
	reticulate	–	√
	raspailiid ectosome	√	√
Ectosomal styles			
	long thin styles	√	√
	short thin styles	√	√
Choanosomal megascleres			
	thick short styles	√	–
	oxeas	–	√
Polyactines			
	equiangular	√	√
	sagittal	√	√
	all cladi spined	√	–
	only basal cladus spined	√	√
	swollen apices	√	–
Trichodragmata			
	present	–	√

Possibly, the position of the polyactines in the skeleton is different in the two genera: usually a basal or central concentration of these spicules in *Cyamon* and more peripheral or scattered throughout in *Trikentrion*, but more observations are necessary to confirm this feature.

The isolated occurrence of such unusual polyactine spicules in two genera that are otherwise likely to belong to monactine raspailiids could be interpreted as support for Uriz and Maldonado’s (1995) hypothesis – based on research of crambeid sponges - that monaxone spicules have evolved from ancestral polyaxones by reduction. Circumstantial evidence (different shape and spination and different position in the skeleton) points towards the possibility that the polyactines of the two genera have a different evolutionary origin: *Cyamon* species could have derived their polyactines from styles, or perhaps acanthostyles (as is suggested by the polyactines of *Cyamon spinispinosum*), whereas *Trikentrion* polyactines could have been derived from choanosomal oxeas. This would mean that the two genera do not share a common ancestor not shared also by other raspailiid groups and the subfamily Cyamoninae would then be artificial.

On the basis of the current state of our knowledge, with, for example, a compelling similarity of polyactines of *Cyamon arguinense* sp. n. and *Trikentrion catalina* (compare Figs 6D and 25D, lower right), such a hypothesis lacks sufficient support, and likewise cannot yet be interpreted as support for Uriz and Maldonado’s (1995) theory. Phylogenetic relationships based on DNA sequence information for the present genera are still tentative. Erpenbeck et al. (2007b) confirmed that *Trikentrion flabelliforme* is a member of a restricted Raspailiidae clade, but is not clearly differentiated from other genera. We will have to await further sequence analysis, which could help to answer the questions whether *Cyamon* and *Trikentrion* are non-monophyletic and whether *Cyamon spinispinosum* is really a *Cyamon*.

## Supplementary Material

XML Treatment for Cyamon

XML Treatment for Cyamon vickersii

XML Treatment for Cyamon amphipolyactinum

XML Treatment for Cyamon arguinense

XML Treatment for Cyamon agnani

XML Treatment for Cyamon aruense

XML Treatment for Cyamon koltuni

XML Treatment for Cyamon neon

XML Treatment for Cyamon argon

XML Treatment for Cyamon quinqueradiatum

XML Treatment for Cyamon quadriradiatum

XML Treatment for Cyamon hamatum

XML Treatment for Cyamon (?) spinispinosum

XML Treatment for Trikentrion

XML Treatment for Trikentrion muricatum

XML Treatment for Trikentrion laeve

XML Treatment for Trikentrion flabelliforme

XML Treatment for Trikentrion helium

XML Treatment for Trikentrion catalina

XML Treatment for Trikentrion africanum

## References

[B1] Alcolado P (1994) List of sponges found in Cuba. Mimeographed list (presumably unpublished): 1–4.

[B2] Alvarez B Hooper JNA (2009) Taxonomic revision of the order Halichondrida (Porifera: Demospongiae) from northern Australia. Family Axinellidae. The Beagle, Records of the Museums and Art Galleries of the Northern Territory 25: 17-42.

[B3] Arndt W (1927) Kalk- und Kieselschwämme von Curaçao. Bijdragen tot de Dierkunde 25: 133-158.

[B4] Boury-Esnault N (1973) Résultats Scientifiques des Campagnes de la ‘Calypso’. Campagne de la ‘Calypso’ au large des côtes atlantiques de l’Amérique du Sud (1961–1962), I, 29. Spongiaires. Annales de l’Institut océanographique 49 (Supplement 10): 263–295.

[B5] Bowerbank JS (1862) On the Anatomy and Physiology of the Spongiadae, Part II. Philosophical Transactions of the Royal Society 152 (2): 747-829.

[B6] Bowerbank JS (1864) A monograph of the British Spongiadae, Volume 1. Ray Society, London, xx + 290 pp.

[B7] Brown RW (1985) Composition of scientific words. Revised edition, 1956. Reprinted 1985. Smithsonian Institution Press, Washington, D.C., 882 pp.

[B8] Burton M (1948) Marine sponges of Congo coast. Institut royal colonial Belge Bulletin des Séances 19 (3): 753-758.

[B9] Burton M (1956) The sponges of West Africa. Atlantide Report (Scientific Results of the Danish Expedition to the Coasts of Tropical West Africa, 1945–1946, Copenhagen) 4: 111–147.

[B10] Burton M Srinivasa Rao H (1932) Report on the shallow-water marine sponges in the collection of the Indian Museum, Part I. Records of the Indian Museum 34: 299-358.

[B11] Capon RJ Macleod JK Scammels PJ (1986) The trikentrins: novel indoles from the sponge *Trikentrion flabelliforme*. Tetrahedron 42 (23): 6545-6550. doi: 10.1016/S0040-4020(01)88117-5

[B12] Carter HJ (1875) Notes introductory to the study and classification of the Spongida. Part II. Proposed classification of the Spongida. Annals and Magazine of Natural History (4) 16 (92): 126–145, 177–200.

[B13] Carter HJ (1876) Descriptions and figures of deep-sea sponges and their spicules, from the Atlantic Ocean, dredged up on board H.M.S.‘Porcupine’, chiefly in 1869 (concluded). Annals and Magazine of Natural History (4) 18: 226–240, 307–324, 388–410, 458–479. doi: 10.1080/00222937608682035

[B14] Carter HJ (1879) Contributions to our knowledge of the Spongida. Annals and Magazine of Natural History (5) 3: 284–304, 343–360. doi: 10.1080/00222937908562401

[B15] Carter HJ (1880) Report on specimens dredged up from the Gulf of Manaar and presented to the Liverpool Free Museum by Capt. W.H. Cawne Warren. Annals and Magazine of Natural History (5) 6: 35–61, 129–156. doi: 10.1080/00222938009458893

[B16] Carter HJ (1882) New sponges, observations on old ones, and a proposed new group. Annals and Magazine of Natural History (5) 10: 106–125. doi: 10.1080/00222938209459681

[B17] Carter HJ (1883) Contributions to our knowledge of the Spongida. Annals and Magazine of Natural History (5) 12 (71): 308–329.

[B18] Dendy A (1905) Report on the sponges collected by Professor Herdman, at Ceylon, in 1902. In: Herdman WA (Ed) Report to the Government of Ceylon on the Pearl Oyster Fisheries of the Gulf of Manaar, 3 (Supplement 18): 57–246.

[B19] Dendy A (1922) Report on the Sigmatotetraxonida collected by H.M.S. ‘Sealark’ in the Indian Ocean. Reports of the Percy Sladen Trust Expedition to the Indian Ocean in 1905, Volume 7. Transactions of the Linnean Society of London (2) 18 (1): 1–164.

[B20] Dendy A (1924) Porifera. Part I. Non-Antarctic sponges. Natural History Report. British Antarctic (Terra Nova) Expedition 1910 (Zoology) 6 (3): 269–392.

[B21] Dickinson MG (1945) Sponges of the Gulf of California. Reports on the collections obtained by Alan Hancock Pacific Expeditions of Velero III off the coast of Mexico, Central America, South America, and Galapagos Islands in 1932, in 1933, in 1934, in 1935, in 1936, in 1937, in 1939, and 1940. The University of Southern California Press, Los Angeles, 55 pp.

[B22] Ehlers E (1870) Die Esper’schen Spongien in der zoologischen Sammlung der K. Universität Erlangen. ET Jacob, Erlangen, 36 pp.

[B23] Ellis J (1766) On the nature and formation of sponges: in a letter from John Ellis, Esquire, F.R.S. to Dr. Solander, F.R.S. Philosophical Transactions (1765) 55: 280–289.

[B24] Erpenbeck D Duran S Rützler K Paul V Hooper JNA Wörheide G (2007a). Towards a DNA taxonomy of Caribbean demosponges: A gene tree reconstructed from partial mitochondrial CO1 gene sequences supports previous rDNA phylogenies and provides a new perspective on the systematics of Demospongiae. Journal of the Marine Biological Society of the United Kingdom 87: 1563-1570.

[B25] Erpenbeck D List-Armitage S Alvarez B Degnan BM Wörheide G Hooper JNA (2007b) The systematics of Raspailiidae (Demospongiae: Poecilosclerida: Microcionina) re-analysed with a ribosomal marker. Journal of the Marine Biological Association of the United Kingdom 87: 1571-1576. doi: 10.1017/S0025315407058201

[B26] Esper ECJ (1794) Die Pflanzenthiere in Abbildungen nach der Natur mit Farben erleuchtet, nebst Beschreibungen. Zweyter Theil. Raspe, Nürnberg, 303 pp.

[B27] Gómez P Carballo JL Vazquez LE Cruz JA (2002) New records for the sponge fauna (Porifea: Demospongiae) of the Pacific coast of Mexico (eastern Pacific Ocean). Proceedings of the Biological Society of Washington 115 (1): 223-237.

[B28] Gray JE (1867) Notes on the arrangement of sponges, with the descriptions of some new genera. Proceedings of the Zoological Society of London 1867 (2): 492-558.

[B29] Hentschel E (1912) Kiesel- und Hornschwämme der Aru- und Kei-Inseln. Abhandlungen herausgegeben von der Senckenbergischen naturforschenden Gesellschaft 34 (3): 293-448.

[B30] Higgin T (1877) Description of some sponges obtained during a cruise of the steam-yacht ‘Argo’ in the Caribbean and neighbouring seas. Annals and Magazine of Natural History (4) 19: 291–299. doi: 10.1080/00222937708682143

[B31] Holthuis LB (1995) 1820–1958 Rijksmuseum van Natuurlijke Historie. Karstens Drukkers BV, Leiden, 171 pp.[in Dutch]

[B32] Hooper JNA (1991) Revision of the family Raspailiidae (Porifera: Demospongiae), with description of Australian species. Invertebrate Taxonomy 5 (6): 1179-1418. doi: 10.1071/IT9911179

[B33] Hooper JNA (2002) Family Raspailiidae Hentschel, 1923. In: Hooper JNA Van Soest RWM (Eds). Systema Porifera. Guide to the classification of sponges,1. Kluwer Academic/Plenum Publishers, New York, Boston, Dordrecht, London, Moscow: 469-510.

[B34] Hooper JNA Van Soest RWM(Eds) Systema Porifera. Guide to the classification of sponges. Kluwer Academic/Plenum Publishers, New York, Boston, Dordrecht, London, Moscow, xxi + 1817 pp.

[B35] Lamarck JBP De (1814) Sur les polypiers empâtés. Suite du mémoire intitulé: Sur les polypiers empâtés. Suite des éponges. Annales du Muséum national d’histoire naturelle Paris 20 (6): 294–312, 370–386, 432–458.

[B36] Laubenfels MW De (1930) The sponges of California. (Abstracts of dissertations for the degree of doctor of philosophy). Stanford University Bulletin (5) 5 (98): 24–29.

[B37] Laubenfels MW De (1932) The marine and fresh-water sponges of California. Proceedings of the United States National Museum 81 (2927): 1-140. doi: 10.5479/si.00963801.81-2927.1

[B38] Laubenfels MW De (1936) A discussion of the sponge fauna of the Dry Tortugas in particular and the West Indies in general, with material for a revision of the families and orders of the Porifera. Carnegie Institute of Washington (Tortugas Laboratory Paper N° 467) 30: 1–225.

[B39] Laubenfels MW De (1950) The Porifera of the Bermuda Archipelago. Transactions of the Zoological Society of London 27 (1): 1-154. doi: 10.1111/j.1096-3642.1950.tb00227.x

[B40] Lee WL Elvin DW Reiswig HM (2007) The sponges of California. A guide and key to the marine sponges of California. Monterey Bay Sanctuary Foundation, Monterey, CA, USA, × + 130 + P266.

[B41] Lendenfeld R Von (1888) Descriptive catalogue of the sponges in the Australian Museum, Sidney. Taylor & Francis, London, xiv + 260 pp.

[B42] Linnaeus C (1759) Systema naturæ per regna tria naturæ, secundum classes, ordines, genera, species, cum characteribus, differentiis, synonymis, locis. Tomus II. Editio decima, reformata. Laurentii Salvii, Holmiæ, 1–4 + 825–1384.

[B43] Linnaeus C (1767) Systema naturae sive regna tria naturae, secundum classes, ordines, genera, species, cum characteribus, differentiis, synonymis, locis. Laurentii Salvii, Holmiae 12 (1, pt 2): 533–1327.

[B44] Linnaeus C (1788) Systema Naturae per regna tria naturae, secundum classes, ordines, genera, species, cum characteribus differentiis, synonymis, locis. Editio decima tertia, aucta reformata. G.E. Beer, Lipsiae. Cura J.F. Gmelin 1(5–6): 2225-3910.

[B45] Little FJ (1963) The sponge fauna of the St. George’s Sound, Apalache Bay, and Panama City Regions of the Florida Gulf coast. Tulane Studies in Zoology 11 (2): 31-71.

[B46] Luke SR (1998) Catalogue of the benthic invertebrate collections of the Scripps Institute of Oceanography. Porifera. SIO Reference Series, 98–6: i–iv, 1–31.

[B47] Montagu G (1818) An essay on sponges, with descriptions of all the species that have been discovered on the coast of Great Britain. Memoirs of the Wernerian Natural History Society 2 (1): 67-122.

[B48] Morrow CC Picton BE Erpenbeck D Boury-Esnault N Maggs, CA Allcock AL (2012) Congruence between nuclear and mitochondrial genes in Demospongiae: A new hypothesis for relationships within the G4 clade (Porifera: Demospongiae). Molecular Phylogenetics and Evolution 62: 174-190. doi: 10.1016/j.ympev.2011.09.016 22001855

[B49] Mothes B Santos CP Campos MA (2004) *Timea bioxyasterina* sp. n., a new species from the Northeastern coast of Brazil (Demospongiae, Hadromerida). Zootaxa 443: 1-8.

[B50] Pallas PS (1766) Elenchus zoophytorum sistens generum adumbrations generaliores et specierum cognitarum succinctas descriptiones cum selectis auctorum synonymis. P van Cleef, The Hague, 451 pp.doi: 10.5962/bhl.title.6595

[B51] Schmidt O (1862) Die Spongien des adriatischen Meeres. Wilhelm Engelmann, Leipzig, viii + 88 pp.

[B52] Seba A (1734–1765) Locupletissimi rerum naturalium thesauri, Volume 3. Janssonio-Waesbergios, Wetstenium & Smith, Amsterdam, 115 pls.

[B53] Sim CJ Bakus GJ (1986) Marine sponges of Santa Catalina Island, California. Occasional Papers of the Allan Hancock Foundation 5: 1-23.

[B54] Sollas WJ (1879) On *Plectronella papillosa*, a new genus and species of echinonematous sponge. Annals and Magazine of Natural History (5) 3: 17–27. doi: 10.1080/00222937908682472

[B55] Spalding MD Fox HE Allen GR Davidson H Ferdaña ZA Finlayson M Halpern BS Jorge AJ Lombana A Lourie SA Martin KD McManus E Molnar J Recchia CA Robertson J (2007) Marine ecoregions of the world: a bioregionalization of coastal and shelf areas. Bioscience 57 (7): 573-583. doi: 10.1641/B570707

[B56] Stephens J (1921) Sponges of the coasts of Ireland, II. The Tetraxonida (concluded). Scientific Investigations of the Fisheries Branch, Department of Agriculture for Ireland 1920 (2): 1-75.

[B57] Thomas PA (1973) Marine Demospongiae of Mahé Island in the Seychelles Bank (Indian Ocean). Annales du Musée royal de l’Afrique central, Sciences zoologiques 203: 1-96.

[B58] Topsent E (1889) Quelques spongiaires du Banc de Campêche et de la Pointe-à-Pître. Mémoires de la Société zoologique de France 2: 30-52.

[B59] Topsent E (1894) Application de la taxonomie actuelle à une collection de spongiaires du Banc de Campêche et de la Guadeloupe décrite précédemment. Mémoires de la Société zoologique de France 7: 27-36.

[B60] Topsent E (1904) Spongiaires des Açores. Résultats des campagnes scientifiques accomplies par le Prince Albert I. Monaco 25: 1-280.

[B61] Topsent E (1928) Spongiaires de l’Atlantique et de la Méditerranée provenant des croisières du Prince Albert ler de Monaco. Résultats des campagnes scientifiques accomplies par le Prince Albert I, Monaco 74: 1-376.

[B62] Topsent E (1931) Éponges de Lamarck conservées au Muséum de Paris. Archives du Muséum national d’histoire naturelle, Paris (6) 5: 1–56.

[B63] Topsent E (1932) Éponges de Lamarck conservées au Muséum de Paris. Deuxième partie. Archives du Muséum national d’histoire naturelle, Paris (6) 8: 61–124.

[B64] Topsent E (1933) Éponges de Lamarck conservées au Muséum de Paris. Fin. Archives du Muséum national d’histoire naturelle, Paris (6) 10: 1–60.

[B65] Uriz MJ Maldonado M (1995) A reconsideration of the relationship between polyaxonid and monaxonid spicules in Demospongiae: new data from the genera *Crambe* and *Discorhabdella* (Porifera). Biological Journal of the Linnean Society 55: 1-15. doi: 10.1016/0024-4066(95)90025-X

[B66] Van Soest RWM (1984) Marine sponges from Curaçao and other Caribbean localities, Part III. Poecilosclerida. Studies on the Fauna of Curaçao and other Caribbean Islands 62 (191): 1-173.

[B67] Van Soest RWM (1993) Distribution of sponges on the Mauritanian continental shelf. In: Wolff WJ van der Land J Nienhuis PH De Wilde PAWJ (Eds). Ecological Studies in the Coastal Waters of Mauritania. Hydrobiologia 258: 95–106.

[B68] Van Soest RWM (1994a) Chapter 6.1. Sponges of the Seychelles. Pp 65–74, In: Van der Land J (Ed) Oceanic Reefs of the Seychelles. Netherlands Indian Ocean Programme, National Museum of Natural History, Leiden, 192 pp.

[B69] Van Soest RWM (1994b). Demosponge distribution patterns. In: Van Soest RWM Braekman JC Van Kempen TMG (Eds). Sponges in time and space. Balkema, Rotterdam: 213-220.

[B70] Van Soest RWM (2009) New sciophilous sponges from the Caribbean (Porifera: Demospongiae). Zootaxa 2107: 1-40.

[B71] Van Soest RWM Cleary DFR De Kluijver MJ Lavaleye MSS Maier C Van Duyl FC (2007) Sponge diversity and community composition in Irish bathyal coral reefs. Contributions to Zoology 76 (2): 121-142.

[B72] Van Soest RWM Boury-Esnault N Hooper JNA Rützler K de Voogd NJ Alvarez de Glasby B Hajdu E Pisera AB Manconi R Schoenberg C Janussen D Tabachnick KR Klautau M Picton B Kelly M (2012) World Porifera database. Available from http://www.marinespecies.org/porifera [accessed on 20 July 2012]

[B73] Van Soest RWM Boury-Esnault N Vacelet J Dohrmann M Erpenbeck D De Voogd NJ Santodomingo N Vanhoorne B Kelly M Hooper, JNA (2012) Global diversity of sponges (Porifera). PLoSONE 7 (4): e35105, 23 pp. 10.1371/journal.pone.0035105 PMC3338747 22558119

[B74] Wolff WJ Van der Land J Nienhuis PH De Wilde PAWJ (Eds) (1993) Ecological studies in the coastal waters of Mauritania. (Developments in Hydrobiology 86). Hydrobiologia 258: × + 222 pp.

